# The perception and experience of dignity in the care of older adults in nursing homes: A Meta-aggregation protocol

**DOI:** 10.1371/journal.pone.0351774

**Published:** 2026-07-21

**Authors:** Chunxiang Li, ZhengKe Li, Jiayi Xu, Rongyu Hua

**Affiliations:** 1 School of Nursing, Henan University of Science and Technology, Luoyang, Henan, China; 2 Faculty of nursing, Chiang Mai University, Chiang Mai, Thailand; 3 College of Earth Sciences, Guilin University of Technology, Guilin, Guangxi, China; Cairo University Faculty of Nursing, EGYPT

## Abstract

**Background:**

The concept of dignity in the context of nursing home care for older adults remains vague, hindering further research in this area.

**Aim:**

This systematic review aims to conceptualize the characteristics of dignity in nursing home care for older adults, with attention to Chinese cultural perspectives.

**Methods:**

The six international databases SCOPUS, PubMed, CINAHL, Embase, Ovid, and PsycInfo, and one Chinese database CNKI will be searched from inception to 31st August 2025, and eligible studies will be primarily evaluated. The literature selected will focus on primary studies that explore older adults’ perceptions of dignity who have resided in the nursing home for at least one month, encompassing their experiences and the factors influencing dignity. This review will draw exclusively on peer-reviewed journal articles, with no language restrictions applied. Grey literature — including dissertations, theses, conference proceedings, and institutional reports — will not be considered for inclusion. The systematic review will adhere to the PRISMA 2020 guidelines and will be registered with PROSPERO. Three reviewers (a lead researcher, a graduate student assistant, and a non-nursing assistant) will primarily screen the titles and abstracts separately. And two professional reviewers (the lead researcher and the graduate student assistant) continue to select studies and extract data independently following the predesigned data extraction forms. Any discrepancies will be documented and resolved by a third external reviewer until consensus is reached. The quality of the included literature will be assessed using the Joanna Briggs Institute (JBI) critical appraisal tools, and the findings will be synthesized using the meta-aggregation approach.

**Expected outcomes:**

This review is expected to identify key themes across two domains: inherent dignity (satisfying physical needs) and social dignity (individuality, identity and independence). It will also identify the key factors influencing dignity, including personal factors (functional status), organizational factors (staff attitudes, organizational ethical environment), and sociocultural factors (Chinese cultural norms such as collectivism). The results of the dignity in care will help healthcare providers implement dignified care in nursing homes.

## Introduction

Global population aging is advancing at an unprecedented pace. China stands out as one of the most prominent nations within this global demographic trend, possessing the world’s largest and fastest-growing elderly population [1,2]. By 2024, China is projected to have 220.23 million individuals aged 65 and above, representing 15.6% of the total population [2]. The global aging trend has intensified the burden of chronic disease and multimorbidity, escalating the demand for long-term care services. In addition, due to smaller family sizes, the one-child policy, and the aging of China’s baby-boom generation, many urban older adults are choosing or being placed in institutions as their adult children become increasingly unavailable due to work responsibilities [3]. These nursing homes have become a pragmatic choice for families, providing comprehensive nursing care for frail older adults with physical and/or cognitive impairments, offering support with activities of daily living (ADLs), and alleviating the care burden on families who lack the capacity to provide adequate care [4].

However, some evidence has shown that undignified care in nursing homes threatens the dignity of older adults and leads to negative outcomes. For instance, violations of dignity: such as neglect, overlooking individual needs, and abuse may result in negative emotional outcomes, including depression and anxiety, as well as increased symptom burden and psychological distress, ultimately leading to a profound sense of hopelessness among older adults [5–11].

Dignity in care has been extensively studied across hospitals, nursing homes, and community settings. However, its meaning varies across cultural and contextual backgrounds, leading to a lack of consensus in its conceptualization. Particularly in nursing home settings, the ambiguity in the conceptualization of older adults’ dignity hinders nursing staff from implementing standardized care practices to maintain dignity. Synthesizing knowledge related to dignity in care for older adults in nursing homes may help to identify key themes and domains, and guide nursing staff in implementing dignified care practices.

According to Jacobson and Høy, dignity comprises two dimensions: inherent dignity and social dignity [12–14]. Inherent dignity refers to the fundamental right of every person, regardless of age, gender, cultural background, or social status [15,16]. It is an inalienable quality that belongs to each individual by virtue of their humanity, often referred to as human dignity [14,17]. Social dignity is generated through interactions between individuals, collectives, and societies, shaped by individual differences and idiosyncrasies [13,14,18]. It is experienced, bestowed, or earned through social interactions, and is scalable, measurable, contingent, comparative, and contextual in nature [13,19].

As population aging intensifies, nursing homes in China have increasingly recognized the importance of dignity in care. Although studies on dignity in nursing home care exist in both Western and Chinese contexts, no systematic review has synthesized qualitative evidence incorporating the Chinese cultural context. Given China’s distinct cultural values, such as collectivism, an integrated synthesis of Western and Chinese perspectives is needed to develop a more comprehensive understanding of dignity among older adults in nursing homes.

Therefore, this systematic review aims to synthesize qualitative evidence through meta-aggregation to explore the perceptions and experiences of dignity among older adults in nursing homes. Subsequently, this review will apply Jacobson’s dignity-theoretical framework to organize and interpret the synthesized findings by integrating Western and Chinese cultural perspectives. Firstly, identify key themes through the lenses of inherent and social dignity. Secondly, organize influencing factors according to actors and environment components of the framework. The findings are expected to contribute new knowledge on dignified care for older adults and inform the development of strategies to improve their experiences in nursing homes.

## Materials and methods

### Research questions

What are the perceptions and experiences of dignity among older adults in nursing homes across Western and Chinese cultural contexts?

What are the personal, organizational, and sociocultural factors that influence dignity among older adults in nursing homes across Western and Chinese cultural contexts?

### Objectives

1. To identify the perceptions and experiences of dignity in care among older adults living in nursing homes.
2. To categorize the personal, organizational, and sociocultural factors that influence dignity in care from the perspective of older adults living in nursing homes.

### Design

The knowledge of Qualitative studies (or the qualitative part of mixed methods studies) related to perception,views or viewpoints, perspective, feelings, attitudes, interpretation, cognition, meaning, and experience of dignity in the database will be collected and synthesized by two researchers. This systematic review protocol is reported in accordance with the Preferred Reporting Items for Systematic Review and Meta-Analysis Protocols 2015 statement [20] (see S1 File), which is specifically designed for protocols. The completed review will be reported in accordance with the 2020 PRISMA guidelines [21].

### Eligibility criteria

The literature will follow the PICo framework to synthesize the literature [22,23]. This framework can serve as the inclusion criteria for the literature (see S1 Table).

### Types of studies

This review will include only qualitative studies that report primary data [24]. The qualitative research approach includes grounded theory, phenomenology,case study,action research, ethnography, and other qualitative research or mixed methods containing qualitative research components. The data collection can include not only interviews and focus groups but also observations. The data analysis includes content analysis, thematic analysis, narrative analysis and general descriptive analysis. Studies published between 1966 (Setting up the database) and 31st August 2025.

### Types of settings and participants

**Population**: Older adults living in nursing homes without severe Alzheimer’s disease or psychological disease. These older adults can understand questions and provide reliable self-reported data. They have the ability to give valid consent that will not violate standard ethical guidelines.

**Phenomenon of Interest:** The review will include qualitative studies exploring (Experience/understanding/views/viewpoints/perspectives/attitudes/perception/feelings/cognition/interpretation/ meaning) of dignity, as well as studies using related terms such as “dignified”or “dignifying” and those published in Chinese.

**Context:** Nursing homes include care homes,long-term care facilities, aged care homes, residential care facilities and other institutions that provide 24-hour assisted care for older adults.

### Inclusion criteria

All studies must explore the experience/understanding/views/ viewpoints /perspective/perception/feeling/cognition/ attitudes/interpretation/ meaning of dignity in nursing home care from the older adults’ perspective. The participants are older adults who have lived there for more than one month.

### Exclusion criteria

Non-primary study: such as a systematic review.

The protocol of the literature.

Grey literature: including theses and dissertations (such as ProQuest), grey reports (such as OpenGrey), and non-peer-reviewed documents and conference proceedings (such as Google Scholar). Grey literature will be excluded from this review. It accounts for a small portion of included studies and rarely affects the results and conclusions, lacks reproducibility and may potentially introduce unpredictable bias due to differ systematically from peer-reviewed literature in ways [25,26].

The perspectives from other individuals except the older adults.

The article related to Long-term care facilities for older adults with serious diseases.

### Search strategy

The search for this title dignity for older adults will be carried out in six International databases and one Chinese database, SCOPUS, PubMed, CINAHL, Embase,Ovid and PsycInfo from the year 1966(set up database) to 31st August 2025. And the CNKI database from the year unlimited to the year 31st August 2025. We will use the PICo framework to identify keywords in the review question (see S2 Table). Population-older adults, conducted a little literature search using the terms “aged”, “older adults”,“the elderly”,“aging”,“geriatric”,“Gerontology”, “senior” respectively in all fields to search. To include data on a wider range of phenomenon, we choose the key words “Mesh word: dignity/dignified/dignifying” to broadly search for the Interest phenomenon. Context: Nursing home/long-term care facilities/ institutions/care homes/aged care homes/Residential care facilities. An example of the Pubmed search as follows: ((((digni*[Title])) AND ((aged[MeSH] OR “older adults”[Title/Abstract] OR “the elderly”[Title/Abstract] OR aging[Title/Abstract] OR geriatric[Title/Abstract] OR Gerontology[Title/Abstract] OR senior[Title/Abstract]))) AND (((“Nursing Homes”[Mesh] OR “Long-Term Care”[Mesh] OR “Homes for the Aged”[Mesh]) OR (“nursing home”[Title/Abstract] OR “long-term care”[Title/Abstract] OR “residential aged care”[Title/Abstract] OR “skilled nursing facility”[Title/Abstract] OR “aged care facility”[Title/Abstract] OR “care home”[Title/Abstract] OR “residential care”[Title/Abstract])))) AND ((“1966/01/01”[Date – Publication]: “2025/08/31”[Date – Publication])).

The review team consists of three members. The primary researcher is a nursing PhD candidate who has received formal training in systematic review methodology and obtained certification from the Joanna Briggs Institute(JBI) training program. The second member is a nursing graduate student assitant who completed evidence-based practice coursework during the master’s learning. The third member is a non-nursing assistant with a Bachelor’s degree,trained in database searching and screening procedures. The primary researcher and the nursing graduate student assistant will be jointly responsible for literature searching and study selection, while the non-nursing assistant will conduct the initial screening only. Prior to the review process, all reviewers will receive standardized training in JBI methodology and the use of critical appraisal tools. Pilot testing of the search strategy, study selection, data extraction, and critical appraisal will be conducted to ensure consistency among reviewers. Study selection and critical appraisal are performed independently by two reviewers (the primary researcher and the nursing graduate student assistant), and any disagreements will be resolved through discussion or, if necessary, consultation with a third reviewer.

## Study records

### Data management, selection and collection process

Search results will be imported into EndNote X8 Management Software, where duplicate entries will be removed by three people. One non-nursing assistant and two researchers (the lead researcher and the graduate student assistant) are for separating initial screenings of papers. Re-reading the literature and incorporating the final selected literature are the responsibility of two researchers(the researcher and the graduate student assistant) to increase trustworthiness and eliminate bias. All three reviewers are screening the literature based on the Eligibility criteria. The studies will be conducted in three stages. In case of inconsistencies in final literature inclusion, especially during the last stage when the full texts are checked, a third external expert will be consulted to resolve the disagreement.

**The first stage involves** conducting a search of the pertinent databases to identify relevant titles and saving them in the reference manager-EndNote X8. A total of two individuals (the lead researcher and the graduate student assistant) are responsible for the comprehensive search of the relevant literature based on the search strategy, while a further three individuals (the lead researcher, the graduate student assistant, and a non-nursing assistant) are tasked with the preliminary screening of the literature according to the specified titles (if the title did not clarify the phenomenon, save it first, then after the abstract reading, decide delete or save it). In order to uncover the studies most relevant to the review, the title and the key words included the dignity or dignified care or dignifying. **In the second stage**, the two reviewers (the lead researcher and the graduate student assistant) will screen the abstracts of the selected titles based on the inclusion criteria,respectively. The final selected literature will go to the third stage. Any disagreements will be resolved through discussion, and if consensus cannot be reached, an external expert will be consulted. For non-English and non-Chinese literature, the translation tool DeepL(27)will be used to translate titles and abstracts prior to screening. To ensure translation accuracy, a bilingual expert or postgraduate student proficient in the source language will be consulted for verification. **In the third stage**,two researchers(the researcher and the graduate student assistant) continue to review the full text and synthesize the information following the form as title,author,year,type and aim of research, research outcome whether manifesting the older adults’ perception about the dignity in nursing home care. If some articles are not available in the database, the reviewers will try to contact the authors to get them and use other relevant database sources to find them. If there is no response to the request for the article within one week, the unavailable article will be excluded. The reason for its deletion will be noted. For the non-English and Chinese literature will be translated to the familiar language to synthesize the information. The study will follow the PRISMA 2020 flow diagram [21] to report the screening results(see S1 Fig).

### Critical appraisal

The objective of critical appraisal in a systematic review is to evaluate the methodological rigor of studies and to identify substantial methodological issues that may compromise the quality of the review findings [28–30]. In the future, the two researchers will use the JBI 10-item checklist [31,32] to evaluate the quality of the articles respectively (see S2 File). The JBI Checklist for Qualitative Research focuses on congruity between philosophical perspective, methodology, and analytical approach, making it particularly suited to appraising the diverse qualitative studies included in this review [31]. Before data collection and analysis, the reviewers will be trained in the JBI tools to ensure they understand each item and assess the literature to the same standard. Following Evans’s [33] approach, we will evaluate the quality of each article using the JBI checklist as follows. The quality of the paper will be ranked as high, medium and low level. If more than 70% items satisfy “yes”, this indicates that a rigorous and robust scientific approach that meets most JBI benchmarks [33], and the literature will be at the high level. If 50%−70% of items satisfy “yes”,this indicates that some flaws do not seriously undermine the overall quality and methodological value of the research, and the literature will be on the middle level. And<50% of items satisfy “yes”, this indicates substantial flaws and poor methodological quality, and the literature can be categorized as the low level. There are two situations that refer to a discussion or a third independent expert for arbitration. First, when there is a discrepancy in the ranking results, the two reviewers will discuss to reach a consensus. If consensus cannot be reached, a third independent external expert will make the final ranking decision. Second, when the literature is rated as low-quality by both reviewers (<50% “yes” on the JBI checklist),it will be retained by default, and excluded only if it contains a critical methodological flaw that directly undermines the credibility of its findings [32]. In cases where the two reviewers disagree on the exclusion decision, a third independent external expert will be consulted for final arbitration. The aim of quality appraisal is to inform the interpretation of findings rather than to necessitate exclusion [32]. All decisions and discussion notes will be documented explicitly.

### Data extraction

The data extraction uses a form including general information and the contents: title, year, author, country, aim, methodology, methods, data analysis method, sample, the population, phenomenon of interest, context, findings, factors influencing dignity. The findings are the verbatim extract of the description or interpretation of the data. Two reviewers extract data separately using the form (see S3 Table). The form will be piloted before use, if it does not clearly manifest the contents, any modifications will be reported and discussed. In qualitative reviews, participants’ quotes are the first-order constructs of data, which are interpreted, stated and understood by the researcher and formed into the second order constructs [28]. Two reviewers will read the raw data on the first and second-order constructs to ensure the findings are grounded in participants’ original experiences. The extracted data will then be imported into NVivo software for further analysis. The extracted findings are the phase of data extraction and the beginning of the data synthesis. An inductive thematic analysis will subsequently be conducted to identify overarching third-order themes.

### Data synthesis

For the sake of transparency of the data synthesis report, the meta-aggregation follows Thomas and Harden’s [34] thematic synthesis of the qualitative research process. And this systematic review protocol was registered in PROSPERO (registration number: CRD420251020581). The whole data analysis and synthesis will be conducted in NVivo software to assist and facilitate researchers in this process. The thematic analysis and synthesis of findings are structured around three stages:

(1) Stage one and two: Coding text and constructing descriptive themes.

Based on the research questions regarding (experience/understanding/views/viewpoints/perspective/perception/feeling/cognition /interpretation/attitudes) of dignity in the care of older adults living in nursing homes, the two reviewers extract and synthesize study findings. Two reviewers independently code these raw data line by line. After coding, the disagreement will be discussed and negotiated and ultimately result in the appropriate “coding” of the phenomenon’s meaning. The inductive approach will be adopted in data extraction and synthesis. Two reviewers will assign group codes to categories based on coding similarity. A category is a group of two or more similar findings that describe a key concept. This description is accompanied by an explanatory statement that conveys the full inclusive meaning of a group of similar findings [31].

(2) Stage three: developing analytical themes

Each category will then be examined and compared with other categories to identify similarities and differences. Similar categories will be grouped into more abstract categories and subsequently synthesized into themes that extend beyond the findings of individual studies to generate a comprehensive understanding of the phenomenon. Guided by Jacobson’s dignity framework [14], the themes are then mapped to the two overarching domains: inherent dignity and social dignity. As noted by Munn [35], the objective of data synthesis or analysis is to create a meaningful representation or set of statements that explicate the phenomenon under study. Each reviewer does this on their own first and then synthesizes the themes as a group. This helped them to discuss more abstract or analytical ideas. The themes emerge naturally and can help address research questions. During data synthesis, the themes should focus on the dignity of older adults in care. Two reviewers should remain sensitive to the research questions and always reflect on the synthesis process.

### Assess confidence from qualitative study synthesis

JBI ConQual approach will be utilized to assess the confidence for the qualitative synthesized findings [36]. The ConQual involves ranking synthesized findings based on dependability and credibility, which separately influence the confidence [36]. Then give an overall score of High, Moderate, Low or Very Low depending on the scoring of the two domains [36] (See S2 and S3 Figs, S3 File).

### The status and timeline of the study

This systematic review is currently in the data searching phase. We will complete it and report findings within half a year, the details of which are as follows:

September 1-September 30,2025: Train the reviewers. Complete data searching and recording. Negotiate the inclusion and exclusion criteria, pilot the screening process, and discuss any problems arising within the screening process.

October 1-October 31,2025: Three reviewers independently conduct title and abstract screening of all retrieved records against the inclusion and exclusion criteria. Disagreements will be resolved through discussion and consensus among the three reviewers.

November 1-November 30,2025: Two reviewers independently conduct full-text screening of all potentially eligible studies. Discrepancies will be resolved through discussion or by consulting a third reviewer if consensus cannot be reached.

December 1-December 31,2025: Two reviewers complete data extraction and appraise the quality of articles. Synthesize the evidence and draft the first findings.

January1- February 28,2026: Conduct manuscript preparation and revision (minimum 3 rounds) by the research team.

### Ethics and dissemination

This systematic review does not involve human participants or animal subjects.

IRB approval for the systematic review protocol is not required.

## Discussion

Based on Jacobson’s dignity theoretical framework [14], this review is expected to generate key themes across two domains. Within Inherent dignity themes related to basic safety needs and physical care needs are anticipated according to Maslow’s Hierarchy of Needs Theory. Within social dignity, themes such as individuality [37], independence [38], and autonomy [38] may emerge. Dignity in care is understood as an outcome influenced by the care environment and the actors who provide care activities in nursing homes [14]. Personal factors such as older adults’ functional status [15,39–41],organizational factors such as the ethical care environment [42,43], and broader sociocultural environmental factors such as collectivism or ageism may influence older adults’ experience and perception of dignity [44–46]. A better understanding of dignity in care from older adults’ perspectives may help identify key themes and behavioral indicators for nursing staff and inform the development of individualized and holistic care strategies to better meet the needs of older adults living in nursing homes.

Furthermore, the findings generated from this study are expected to complement existing knowledge on dignity in care within the Chinese nursing home context. Ultimately, these practice-level findings may contribute to improving both the quality of care and the quality of life for older adults in nursing homes. Compared to previous reviews in this area, those reviews primarily focused on Western cultural contexts and did not incorporate Chinese cultural perspectives on dignity in care [43,47–49]. Moreover, they focused mainly on interpersonal and relational aspects of dignity, with limited attention to institutional-level factors such as care management practices and staff training that are central to the Chinese nursing home setting [43,47–49]. This review is therefore expected to offer novel perspectives on the key findings by adding Chinese cultural literature on dignity in care, and by examining a broader range of dignity characteristics in care in similar experiences, including care collectivism and “harmonious ” culture environment [50,51] and management practices, the healthcare providers’ behaviors, and staff training [52,53].

Despite these advantages, several key challenges exist in implementing the review protocol. First, synthesizing themes and subthemes that accurately represent the original findings is difficult, with the risk that extracted themes may not fully reflect the initial studies’ conclusions. This necessitates comprehensive reviewer training and standardized procedures to ensure consistent extraction quality across all studies. If conflicting findings emerge during the synthesis process, the reviewers will discuss them until a consensus is reached; if persistent disagreements occur, they will be referred to a third expert for final adjudication. Second, as this review synthesizes evidence exclusively from older adults’ perspectives, the findings may not fully capture the complexity of dignity in care as experienced or perceived by other key stakeholders. Future research should incorporate multiple demographic perspectives, including age subgroups (such as youngest-old, ages 65–74 years; middle-old, 75–84 years; and oldest-old, ≥ 85 years) [54], gender(male and female), cultural backgrounds (such as older people who living in urban versus rural nursing homes, and variations across Chinese regional cultures), and care-related characteristics (such as length of stay and functional dependency levels). Given the challenges of directly capturing the perspectives of older adults with dementia or mental health conditions, future studies should also explore dignity in care from the perspectives of healthcare providers (such as nurses and nursing assistants) and family members caring for these vulnerable subgroups to enable comparative analysis and enhance the generalizability of findings.

## Limitations

This review has several limitations. Firstly, language bias. While the DeepL tool [27] will be employed to translate studies into languages beyond English and Chinese, it’s important to acknowledge that machine translation may not fully capture the nuanced meanings of dignity [55]. The Non-English and Non-Chinese studies might be missed entirely if they are not indexed in our selected databases. Secondly, the exclusion of grey literature, encompassing theses, dissertations, and non-peer-reviewed materials, risks introducing selection and publication bias by potentially overlooking relevant studies not indexed in peer-reviewed databases [56]. In particular, excluding theses and dissertations may result in missing emerging research from early career researchers whose findings have not yet reached peer-reviewed publication, further limiting the comprehensiveness of this review. Thirdly, although this review integrates both Western and Chinese cultural perspectives, the findings may not be fully transferable to other cultural contexts, given the unique values and norms that shape experiences and perceptions of dignity within the Chinese cultural setting. Even within Asian cultures, the findings may not be directly applicable to contexts such as Japan or Korea, which have distinct aging demographics, long-term care systems, and cultural values surrounding dignity and elder care that differ meaningfully from those in China. Fourthly, a limitation is that this review will exclude older adults with severe Alzheimer’s disease or significant psychological disorders, as well as perspectives from other relevant stakeholders. This may limit the comprehensiveness of the findings, particularly for individuals who are unable to articulate their experiences. Fifthly, interpretive bias is a potential limitation during coding and theme development. Despite independent analysis by two reviewers, qualitative synthesis inherently involves subjective interpretation, which may be shaped by the reviewers’ disciplinary perspectives and cultural contexts. A further limitation is that the literature search was conducted at a single period in time; as a result, studies published after August 2025 will not be captured.

This protocol has been registered in the International Prospective Register of Systematic Reviews (PROSPERO) (OSF number: CRD420251020581). The findings from this review will be shared via publication in a peer-reviewed journal or presented at academic conferences. No patients or members of the public are involved in the design, conduct, or dissemination plans of this review.

## Conclusions

This Meta-aggregation is expected to provide an overview of qualitative evidence available on older people’s dignity and influencing factors in the nursing home context. The synthesized findings will be created, reflecting the nature of dignity for older adults within inherent and social dignity domains [14]. And individual, organizational and sociocultural factors influencing dignity in care followed up the dimensions of actors and behaviors [14]. The findings of this review are expected to inform practice-level strategies to maintain older adults’ dignity and improve their experiences and perceptions of care in nursing homes, particularly within the Chinese cultural context [31]. Furthermore, the findings may help inform healthcare policy and support the development of standards for dignified care [31]. For nursing practice, nursing staff may adopt a dignity-centered approach that actively maintains older adults’ safety, independence, sense of identity, and autonomy, with culturally sensitive care embedded into routine practice. For nursing education, training curricula should incorporate dignity-focused knowledge, cultural competency, and anti-ageism content as core components. At the policy level, healthcare systems should mandate anti-ageism training as part of professional development and establish dignity as a measurable quality indicator in institutional standards. Future research is recommended to explore and synthesize the meaning of dignity not only from older adults’ perspectives but also from those of nursing staff, family members, administrators and policymakers across diverse cultural settings, to develop a more comprehensive understanding of dignity in care.

## Supporting information

S1 Fig PRISMA Flow Diagram for New Systematic Reviews of Dignity Perception Selection Process. This figure illustrates the dignity literature process of identification, screening, eligibility, and inclusion of studies.
(PDF)

S2 Fig Ranking for dependability. This figure shows the assessment criteria for dependability.
(TIF)

S3 Fig Ranking for Credibility. This figure presents the evaluation criteria for credibility.
(TIF)

S1 File PRISMA-P-2015 Checklist. This file contains the PRISMA-P 2015 checklist used in this review.
(DOCX)

S2 File Checklist for critical and interpretive research. This file presents the criteria used to appraise qualitative studies.
(DOCX)

S3 File ConQual summary of findings example. This file provides an example of the ConQual summary of findings.
(DOCX)

S1 Table PICo Framework. This table describes the population,phenomenon of interest, and context used to guide the review.
(DOCX)

S2 Table Search Strategy. This table presents the detailed database search strategy.
(DOCX)

S3 Table Data Extraction Form. This table shows the form used for extracting data from included studies.
(DOCX)

## Abbreviations

PROSPERO: international prospective register of systematic reviews
PICo: population, phenomenon of interest, context
CASP: critical appraisal skills program
JBI: Joanna Briggs institute
