# Supplementary figures and images for: The perception and experience of dignity in the care of older adults in nursing homes: A Meta-aggregation protocol
Source: PLoS One. 2026 Jul 21;21(7):e0351774. doi: 10.1371/journal.pone.0351774 (PMC13387536; doi:10.1371/journal.pone.0351774)

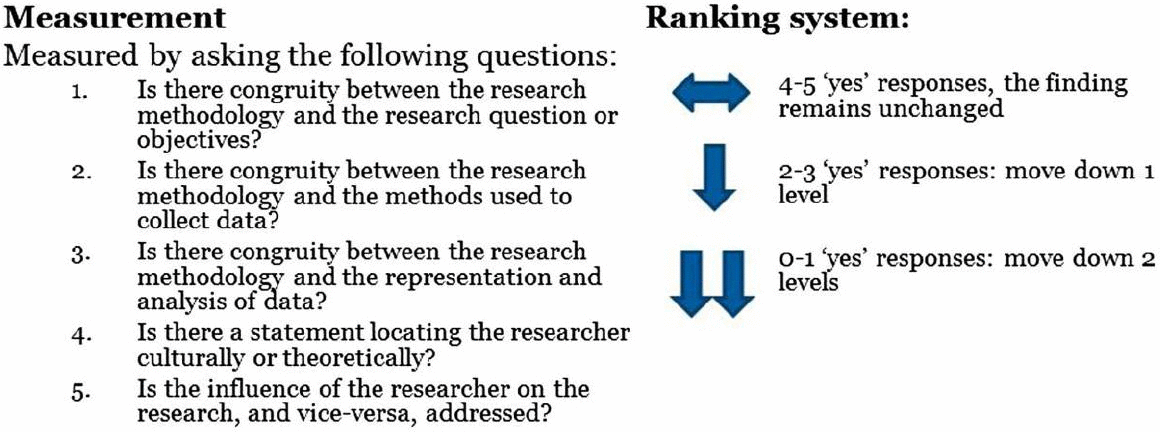

Supplement: S2 Fig — This figure shows the assessment criteria for dependability. (TIF) [file pone.0351774.s002.tif]

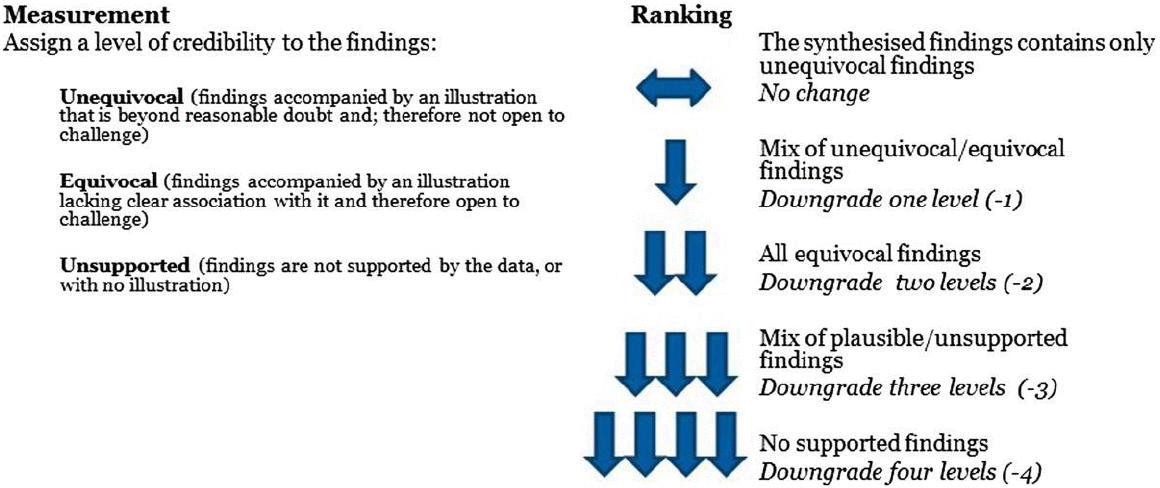

Supplement: S3 Fig — This figure presents the evaluation criteria for credibility. (TIF) [file pone.0351774.s003.tif]
