# Supplementary material for: The perception and experience of dignity in the care of older adults in nursing homes: A Meta-aggregation protocol
Source: PLoS One. 2026 Jul 21;21(7):e0351774. doi: 10.1371/journal.pone.0351774 (PMC13387536; doi:10.1371/journal.pone.0351774)
Supplement: S2 File — This file presents the criteria used to appraise qualitative studies. (DOCX) [file pone.0351774.s005.docx]

**JBI Checklist for critical and interpretive research**


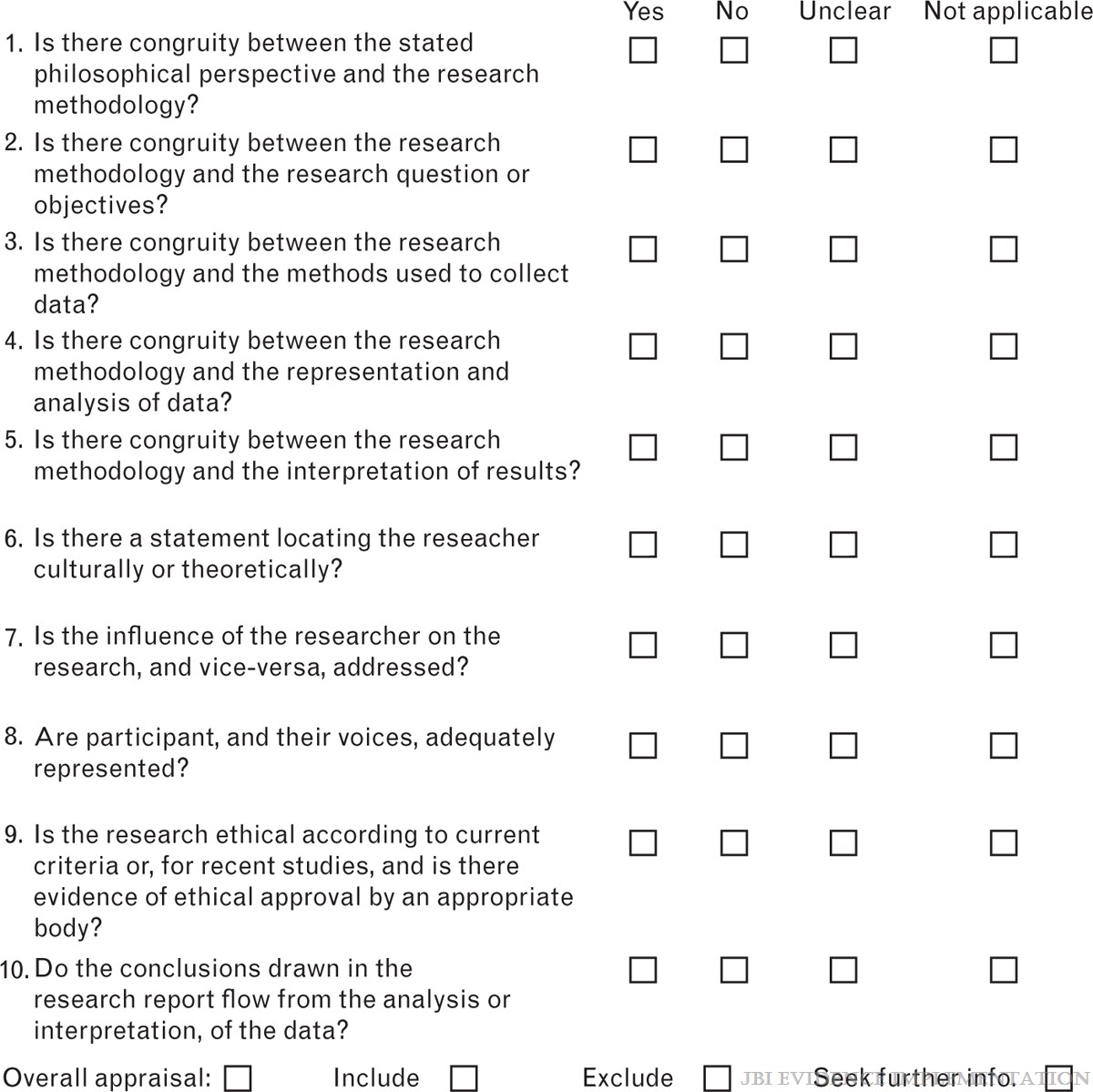


Copyright © 2025 Wolters Kluwer. Published by Lippincott Williams & Wilkins.

Craig L, Zachary M, Kylie P. Qualitative research synthesis: methodological guidance for systematic reviewers utilizing meta-aggregation. Int J Evid Based Healthc. 2015;13:179-87.
