# Supplementary material for: The perception and experience of dignity in the care of older adults in nursing homes: A Meta-aggregation protocol
Source: PLoS One. 2026 Jul 21;21(7):e0351774. doi: 10.1371/journal.pone.0351774 (PMC13387536; doi:10.1371/journal.pone.0351774)
Supplement: S3 File — This file provides an example of the ConQual summary of findings. (DOCX) [file pone.0351774.s006.docx]

**ConQual summary of findings example**

| **Systematic review title:** The perception and experience of dignity in the care of older adults in nursing homes  **Phenomena of interest:** older people’s perception and experience of dignity in care  **Context:** Nursing home | | | | |
| --- | --- | --- | --- | --- |
| **Synthesized finding** | **Type of research** | **Dependability** | **Credibility** | **ConQual score** |
| Theme: Being as an individual | Qualitative | Downgrade 1 level* | Downgrade 1 level** | low |

* Downgraded one level due to common dependability issues across the included primary studies.( For example: half of the studies score 3 out of 5 for statements locating the researcher culturally.)

** Downgraded one level due to a mix of unequivocal and equivocal findings.
