# Supplementary material for: The perception and experience of dignity in the care of older adults in nursing homes: A Meta-aggregation protocol
Source: PLoS One. 2026 Jul 21;21(7):e0351774. doi: 10.1371/journal.pone.0351774 (PMC13387536; doi:10.1371/journal.pone.0351774)
Supplement: S1 Table — This table describes the population,phenomenon of interest, and context used to guide the review. (DOCX) [file pone.0351774.s007.docx]

**PICo Framework**

| **P** | **I** | **Co** |
| --- | --- | --- |
| Older people | Experience/understanding/views/perspective/perception/feeling/cognition / attitudes/interpretation /viewpoints/ meaning of dignity | Nursing home/Long term care facilities/aged care homes/ residential care facilities and related to nursing homes |
