# Supplementary material for: The perception and experience of dignity in the care of older adults in nursing homes: A Meta-aggregation protocol
Source: PLoS One. 2026 Jul 21;21(7):e0351774. doi: 10.1371/journal.pone.0351774 (PMC13387536; doi:10.1371/journal.pone.0351774)
Supplement: S2 Table — This table presents the detailed database search strategy. (DOCX) [file pone.0351774.s008.docx]

**Seach Strategy**

| **P** | **I** | **Co** |
| --- | --- | --- |
| Aging  older  geriatric  Gerontology  senior  the elderly | Dignity/dignified/dignifying | Nursing home  long term facilities/ institutions/  care homes/aged care homes/ Residential care facilities(RCF) |
