# Supplementary material for: The perception and experience of dignity in the care of older adults in nursing homes: A Meta-aggregation protocol
Source: PLoS One. 2026 Jul 21;21(7):e0351774. doi: 10.1371/journal.pone.0351774 (PMC13387536; doi:10.1371/journal.pone.0351774)
Supplement: S3 Table — This table shows the form used for extracting data from included studies. (DOCX) [file pone.0351774.s009.docx]

**Data Extraction Form**

| Title | Year | Country | Author | Aim | Methodology | Method | Data analysis methods | Sample | Population | Phenomena of interest | Context | Findings | factors |
| --- | --- | --- | --- | --- | --- | --- | --- | --- | --- | --- | --- | --- | --- |
|  |  |  |  |  |  |  |  |  |  |  |  |  |  |
