# Supplementary material for: The perception and experience of dignity in the care of older adults in nursing homes: A Meta-aggregation protocol
Source: PLoS One. 2026 Jul 21;21(7):e0351774. doi: 10.1371/journal.pone.0351774 (PMC13387536; doi:10.1371/journal.pone.0351774)
Supplement: Attachment — Submitted filename: Response to Reviewers.docx [file pone.0351774.s011.docx]

Dear Editor and Reviewers,

Thank you for your valuable time and constructive comments on our manuscript. We have carefully addressed all the concerns raised and revised the manuscript accordingly. Below, we provide a point-by-point response to each comment. All changes in the revised manuscript are highlighted in track changes.

We hope that our revisions are satisfactory and look forward to your favourable consideration.

Sincerely,

[Chunxiang Li]

| **Reviewer comments** | | **Response** | **Location in MS** | **Type(Major or Minor)** |
| --- | --- | --- | --- | --- |
| 1 | Resubmitting your figure files. | S2 Fig, S3 Fig | Support information | Major |
| 2 | File naming | S1 Table, S2 Table, S1,S2 S3 Fig, S1,S2 S3 File | Support information | Major |
| 3 | Acknowledgments Section | Have added in the cover letter and deleted in the acknowledgements of manuscript. | Acknowledgments Section | Major |
| 4 | Recommendation to cite specific previously published works | No | No |  |
| **Reviewer comments** | |  |  |  |
| **1-ABSTRACT: 5 mandatory required** | | All five required items have been addressed in the revised abstract. | Line10-34 | Major |
|  | （1）BACKGROUND: State gap clearly. | Clarified the research gap in the Background. | Line11-12 | Major |
|  | （2）AIM :Rewrite, one clear sentence. | Rewritten the Aim as one clear sentence. | Line 13-16 | Major |
|  | （3）METHODS :Add missing items. "Quality will be assessed using JBI. Meta-aggregation will be used for synthesis." | Added the missing quality assessment method. | Lin25-27 | Major |
|  | （4）EXPECTED OUTCOMES: Be specific include key themes, domains, and cultural factors influencing dignity. | Specific in the themes, domains and factors in expected outcomes. | Line28-34 | Major |
| **2. LANGUAGE: Professional language editing required.** | | The language has been improved with the assistance of a PhD colleague who has extensive publication experience. | Whole manuscript | Major |
| **3- The keywords in the abstract have been revised by replacing “older people” with “older adults” to ensure consistency with the title.** | | Thank you for this revision. | Whole manuscript and keywords in the system | Minor |
| **4- Introduction** | |  |  |  |
|  | (1) Restructure the introduction using a clear problem → gap → aim → research question framework. The current version is fragmented and repetitive. | Problem: line 39-60, gap: Line 58-65, aim: Line83-90. Research questions: Line 92-97 | Line 38-97 | Major |
|  | (2) State the research question explicitly. A systematic review protocol must clearly define what it seeks to answer (e.g., “What are the perceptions and experiences of dignity among older adults in nursing homes?”). | There are two specific research questions. | Line93- 97 | Major |
|  | (3) Align the introduction with the abstract. The abstract emphasizes Chinese cultural context, yet the introduction makes no mention of it. This inconsistency must be resolved. | Added a paragraph in the introduction to explicitly discuss the Chinese cultural context (e.g., collectivism, and their influence on dignity in care for older adults). This revision ensures consistency with the abstract. Please see [Section: Introduction, Line 39-57, line75-81]. | Line 39-57,  line 75-81 | Major |
|  | (4) Undergo professional language editing. The manuscript contains frequent grammatical errors, spelling mistakes, and awkward phrasing that obscure meaning and reduce credibility. | (1)Grammarly was used to correct grammatical and spelling errors; (2) A PhD colleague with extensive publication experience copy-edited the manuscript for clarity and readability. | Whole manuscript | Major |
|  | (5) Specify the theoretical framework. Explicitly state which dignity model (e.g., Jacobson, Kitwood) will guide the synthesis and interpretation of findings. | (1) Jacobson dignity theoretical framework help to identify the factors and domains of dignity.(2) The function of this theory has been interpreted in introduction section [Line 66-74, line85-88]. | Line66-74, line85-88 | Major |
|  | (6) Justify the need for a systematic review. Explain why this review is necessary now, what previous reviews have missed, and how this review will contribute new knowledge. | ***Why now:*** Although dignity in care for older adults in nursing homes has received increasing research attention, the evidence has not been systematically synthesized to inform practice . **Line 61-65*.***  ***What previous reviews have missed:*** no systematic review has synthesized qualitative evidence incorporating the Chinese cultural context. **Line 76-81.**  **New knowledge contribution:** generating evidence to inform dignified care strategies for Chinese nursing home settings. **Line 82-90.** | Line 61-65*.*  Line 76-81.  Line 92-90. | Major |
| **5-methodology is clear, appropriate, and well-structured Just 4 Issues to Fix Before Resubmission:** | |  |  |  |
|  | (1) Research Questions – Poorly framed, grammar errors, no PEO structure Rewrite using PEO framework, fix singular/plural, clarify Chinese context | 1. What are the perceptions and experiences of dignity (O) among older adults (P) in nursing homes across Western and Chinese cultural contexts (E)?  2. What personal, organizational, and sociocultural factors (E) influence dignity (O) among older adults (P) in nursing homes across Western and Chinese cultural contexts? | Line 93-97 | Major |
|  | (2) Grey Literature – Too vague ("all qualitative documents found") Specify sources (ProQuest, OpenGrey, Google Scholar) and types (theses, conference proceedings) | Specified the grey literature sources and types in the Exclusion criteria section. | Line142-144 | minor |
|  | (3) Non-English Translation – Unclear process, grammar error ("will be translate") Clarify translation method (e.g., DeepL) and add bilingual verification | We have corrected the grammar error (“will be translate” → “will be translated”). We have also clarified that non-English articles will be translated using DeepL, followed by bilingual verification by a a bilingual expert or postgraduate student proficient in the source language. | Line186-189 | Major |
|  | (4) Language – Pervasive spelling/grammar errors throughout Professional language editing is mandatory. | (1)Grammarly was used to correct grammatical and spelling errors; (2) A PhD colleague with extensive publication experience copy-edited the manuscript for clarity and readability. | Whole manuscript | Major |
| **5- Discussion section needs 4 fixes:** | |  |  |  |
|  | (1)Language – Errors are everywhere ("angles", "culture care", "literatures"). Professional editing is mandatory. | (1)Grammarly was used to correct grammatical and spelling errors; (2) A PhD colleague with extensive publication experience copy-edited the manuscript for clarity and readability. | Whole manuscript | Major |
|  | (2) Tone – You have no results yet. Do not say "will". Use "expected to" or "may" . | Revised the “will” to “expected to” | Line292 | Minor |
|  | (3) Limitations – You forgot language bias, publication bias, and cultural generalizability. Add them. | (1) Language bias: machine translation may not fully capture the nuanced meanings of dignity. Line324-327  (2) Publication bias by potentially overlooking relevant studies not indexed in peer-reviewed databases. Line 327-330.  (3) Not be fully transferable to other cultural contexts.Line 330-333. | Line324-333 | Major |
|  | (4) Missing – Add your PROSPERO number, how you will share findings, and a PPI statement. | Add the PROSPERO number: CRD420251020581. | Line 334-338 | Major |
| **6- Conclusion needs 4 fixes:** | |  |  |  |
|  | (1) Language – "include" → "including", "will be synthesize" → "will be synthesized", "system review" → "systematic review", "nursing home" → "nursing homes". | All four have been corrected. Additionally, we have proofread the entire manuscript to ensure no similar errors remain. | Whole manuscript | Major |
|  | (2) Tone – You have no results yet. Do not say "will" . Say "aims to" or "is expected to" . | Replace future-tense predictions. | Line345 | Major |
|  | (3)Overclaiming – One review cannot produce "guidelines" . Say "inform strategies" instead. | Replace the term “guidelines” with more accurate phrasing “inform strategies” | Line345-347 | Major |
|  | (4) Missing – Add Chinese cultural context and a brief future research sentence. | Add the Chinese cultural context and future research. | Line347-353 | Major |
| **Funding statement and supplementary files are excellent. However, 4 fixes are required:** | |  |  |  |
|  | (1) Author Contributions – Too vague ("screens literature"). Use CRediT taxonomy (Conceptualization, Methodology, Investigation, Writing, Supervision, etc.). Also identify the corresponding author. | Use CRediT taxonomy to describe each author’s contribution. And the corresponding author. | Line 362-367 | Major |
|  | (2)Missing statements – Add Conflict of Interest ("The authors declare no conflicts") and Data Availability ("No new data were generated"). | Add Conflict of Interest and Data Availability. | Line 369-370 | Major |
|  | (3)Abbreviations – Fix CASP (Critical Appraisal Skills Programme), PICo capitalization, and PROSPERO colon. | Abbreviation section has been revised. | Line355-358 | Minor |
|  | (4) Supplementary files – Fix "S 3File" → "S3 File", and correct "Seach Strategy" spelling. | Supplementary files have been revised following the requirements of PLOS ONE. | Line371-391 | Major |
| **7-Reference list:5 fixes are required:** | |  |  |  |
|  | 1. Pick ONE style – Vancouver or APA. Do not mix them. | All references consistently follow Vancouver (Plos One’s　format　 https://journals.plos.org/plosone/article?id=10.1371/journal.pone.0031009#references) throughout the manuscript. | Line 392-516 | Major |
|  | 2. Add DOIs – almost all are missing. This is required in 2025. | Add DOIs for all references where DOIs are available. | Line 392-517 | Major |
|  | 3. Fix incomplete references – Ref 8 ("no-no") is unacceptable. Provide full page numbers. Complete Ref 5, 16, 18. | Ref 8 (previously “no-no”) has been removed following revisions to the Introduction section.  Page numbers have been added where required.  Ref 5 has been corrected to: Ref 18:Gallagher A (2004) Dignity and respect for dignity - Two key health professional values: Implications for nursing practice. Nursing Ethics 11:587-599.<https://doi.org/10.1191/0969733004ne744oa>.  Ref16 has been corrected to: Ref 27: Bettany-Saltikov J, McSherry R(2024) How to do a Systematic Literature Review in Nursing: A Step-by-Step Guide (3rd ed.): Open University Press.  Ref 18 has been corrected to: Ref 29: Aromataris E, Lockwood C, Porritt K, Pilla B, Jordan Z (2024) JBI manual for evidence synthesis.<https://doi.org/10.46658/JBIMES-24-01>. | Ref 5: Line 445-447  Ref 16: Line 472-47  Ref 18: Line477-478 | Major |
|  | 4. Check in-text citations – Refs 12,13,14,16,17,21,22 may not be cited in your manuscript. Add or remove. | Refs 12, 13, 14, 16, 17, 21, 22 have been reviewed. Uncited references have been removed; newly cited references have been added accordingly. | Line 392-517 | Major |
|  | 5. Correct typos – Ref 1 (Oxford), Ref 2 (parentheses), Ref 23 (journal name capitalization). | Ref 1 has been removed following revisions to the Introduction section.  Ref 2 has been revised as:  Ref 2:United Nations (2017) World Population Ageing 2017: Highlights. [https://www.un.org/development/desa/pd/sites/www.un.org.development.desa.pd/ files/files/documents/2020/May/un_2017_worldpopulationageing_report.pdf](https://www.un.org/development/desa/pd/sites/www.un.org.development.desa.pd/files/files/documents/2020/May/un_2017_worldpopulationageing_report.pdf)  Ref 23 has been revised as: Ref 34: Thomas J, Harden A (2008) Methods for the thematic synthesis of qualitative research in systematic reviews. BMC medical research methodology 8:1-10.<https://doi.org/10.1186/1471-2288-8-45>. | Ref 2: Line 396-398  Ref 23: Line 493-495 | Major |
