# Supplementary material for: The perception and experience of dignity in the care of older adults in nursing homes: A Meta-aggregation protocol
Source: PLoS One. 2026 Jul 21;21(7):e0351774. doi: 10.1371/journal.pone.0351774 (PMC13387536; doi:10.1371/journal.pone.0351774)
Supplement: Attachment — Submitted filename: Response_to_Reviewers_auresp_2.docx [file pone.0351774.s012.docx]

Dear Editor and Reviewers,

Thank you for your valuable time and constructive comments on our manuscript again. We have carefully addressed all the concerns raised and revised the manuscript according to the comments. Below, we provide a point-by-point response to each comment. All changes in the revised manuscript are highlighted in track changes.

We hope that our revisions are satisfactory and look forward to your favourable consideration.

Sincerely,

[Chunxiang Li]

|  | **Reviewer comments** | **Response** | **Location in MS** | **Type(Major or Minor)** |
| --- | --- | --- | --- | --- |
| **1** | If you would like to make changes to your financial disclosure, please include your updated statement in your cover letter. Guidelines for resubmitting your figure files are available below the reviewer comments at the end of this letter. | (1)No need to revise the financial disclosure.  (2)Last version we have revised the figures use free NAAS figure tool of PLOS. |  |  |
| **2** | If applicable, we recommend that you deposit your laboratory protocols in protocols.io to enhance the reproducibility of your results. Protocols.io assigns your protocol its own identifier (DOI) so that it can be cited independently in the future. For instructions see: <https://journals.plos.org/plosone/s/submission-guidelines#loc-laboratory-protocols>. Additionally, PLOS ONE offers an option for publishing peer-reviewed Lab Protocol articles, which describe protocols hosted on protocols.io. Read more information on sharing protocols at <https://plos.org/protocols?utm_medium=editorial-email&utm_source=authorletters&utm_campaign=protocols>. | Our manuscript employs a qualitative synthesis methodology and does not involve laboratory protocols. Therefore, depositing on protocols.io is not applicable to our study. |  |  |
| **3** | If the reviewer comments include a recommendation to cite specific previously published works, please review and evaluate these publications to determine whether they are relevant and should be cited. There is no requirement to cite these works unless the editor has indicated otherwise. | We don’t have cited the previously published works. |  |  |
| **4** | Please review your reference list to ensure that it is complete and correct. If you have cited papers that have been retracted, please include the rationale for doing so in the manuscript text, or remove these references and replace them with relevant current references. Any changes to the reference list should be mentioned in the rebuttal letter that accompanies your revised manuscript. If you need to cite a retracted article, indicate the article’s retracted status in the References list and also include a citation and full reference for the retraction notice. | We have carefully reviewed our reference list for completeness and accuracy. No retracted articles were identified among our citations, and no changes to the reference list were made. |  |  |
| Additional Editor Comments | | | | |
| **Abstract:** Your abstract is well written but three essential elements are missing. Please add the following to your abstract before resubmission. | | | | |
| **One.** | Please specify the search timeframe. You need to state the period of the search, for example from inception to 2024 or from 2000 to 2024. | Add the timeframe from inception to 31th August 2025. | Line 18-19 | Minor |
| **Two.** | Please specify your inclusion and exclusion criteria. You need to state the language criteria such as English and Chinese only. You also need to state whether grey literature such as conference proceedings and dissertations will be included or excluded. | We have added the inclusion and exclusion criteria to the abstract as requested:  Language criteria: No language restrictions will be applied (we include all languages, not only English and Chinese).  Grey literature: conference proceedings, dissertations, theses, reports will be excluded. | Line 19-25 | Major |
| **Three.** | Please add transparency and reproducibility elements. | We have added transparency and reproducibility elements to the abstract as requested. This system review will follow the PRISMA 2020 guidelines and will be registered with PROSPERO.  Three reviewers will independently screen, two professional reviewers extract data, and assess study quality.  Disagreements will be resolved by a third expert. JBI critical appraisal tools will help to assess the quality of each article.  Full search strategies will be provided as supplementary materials. | Line26-33 | Major |
| **Introduction:** Your introduction is well written and has several strengths. The problem is clearly stated, the aim is specific, the justification for the review is clear, the theoretical framework of Jacobson and Høy is properly introduced, the Chinese cultural context is explicitly addressed, and the gap in literature is well identified. | | | | |
| **One.** | Please check your in-text citations. Some numbers appear without corresponding references. Ensure every citation number has a full reference in your reference list. | Thank you for your comment on the in‑text citations.  Throughout the manuscript, when multiple consecutive references are cited, they are presented as a range (e.g., [3-6] instead of [3,4,5,6]). These ranges include all numbers in between. Every number appearing in any such range has a full and matching reference in the reference list.  I have double‑checked the entire document, and no citation number is missing its corresponding reference. |  | Minor |
| **Two.** | Your first paragraph is very long. Consider breaking it into two shorter paragraphs for better readability. | We break the first paragraph into two shorter paragraphs: First shorter paragraph introduces the phenomenon of aging in China and nursing home is a necessary choice for older adults. Second paragraph introduces the existing caring problems in nursing home. | Line48-64 | Minor |
| **Three.** | You wrote "hybrid analytical approach" in the last paragraph but you did not explain what this means. Please clarify this term or remove it if not necessary. | Remove the hybrid analytical approach. | Line89. | Minor |
| **Four.** | You stated that this review will use meta-aggregation and then examine findings through Jacobson's framework. Please clarify whether the framework will guide the synthesis or will be applied after the synthesis. needs to be explicit. | The framework just used for the categorizing the themes after the synthesis and help to identify the domains. | Line 89-98 | Major |
| **Method:** Methods section is strong and well developed. The ten issues identified need to be corrected. | | | | |
| **One.** | Date inconsistency. You wrote "31st September 2025" as your search completion date. September has 30 days, not 31. Please correct to 30th September 2025. | The search covered the period from 1966 to 31 August 2025.And the search completed data is 30th September 2025, so I revise the data to keep consistency. | Line178 | Minor |
| **Two.** | Year inconsistency in timeline. In your timeline you wrote October 1-October 31, 2025 and November 1-November 30, 2025 but your search completion date is August 31, 2025. These dates do not match. Please revise your timeline to be consistent with your search completion date. | We would like to respectfully clarify that August 31, 2025 refers to the search end date(the date limit applied to the database search), while September 30, 2025 is the search completion date (the date by which the search will be fully executed and recorded), as noted in your previous comment. Therefore, the October and November phases are fully consistent with the search completion date of September 30, 2025. To avoid any further confusion, we have added explicit labels in the timeline to clearly distinguish the search end date from the search completion date. | Line 346-356 | Major |
| **Three.** | Missing justification for excluding grey literature. You excluded theses, dissertations, and non-peer-reviewed materials. This is a major limitation that may introduce publication bias. Please provide a clear rationale for why grey literature is excluded. | We describe the reason for excluding the grey literature. | Line 157-161 | Minor |
| **Four** | Missing explanation for excluding older adults with Alzheimer's disease. You excluded older adults with severe Alzheimer's or psychological disease. Please explain why this population is excluded as their dignity is also an important concern. | In the current study, we excluded this population primarily for methodological and ethical reasons related to informed consent and response capability. | Line 136-138 | Minor |
| **Five.** | Translation method issue. You stated you will use DeepL for non-English and non-Chinese literature. Machine translation may miss nuanced meanings of dignity. Please add that you will have a bilingual expert verify all translated content. | We would like to clarify that this step has already been included in our manuscript. As stated in the [Methods/Translation section], all translated content from non-English and non-Chinese literature will be initially processed by DeepL and then independently verified by a bilingual expert to ensure the accurate interpretation of nuanced concepts such as "dignity." We have highlighted this in the revised manuscript for clarity. | Line 231-232 | Minor |
| **Six.** | PRISMA version inconsistency. You cited PRISMA 2015 for the protocol and PRISMA 2020 for the review. Please use PRISMA 2020 for both or clarify why different versions are used. | Thank you for your valuable comment. We would like to clarify that different reporting guidelines are applied at different stages of a systematic review.  This study is a systematic review protocol,so its report is in accordance with the PRISMA-P 2015 statement, which is specifically developed for systematic review protocols and has not yet been updated to a 2020 version.  For the completed systematic review in the future, we will follow the PRISMA 2020 guidelines, which represent the most recent standard for reporting systematic reviews.  Therefore, the use of PRISMA-P 2015 for the protocol and PRISMA 2020 for the final review is appropriate and consistent with current methodological recommendations. | Line119-121 | Minor |
| **Seven.** | Missing sample of search strategy. You provided Table 2 but did not show the actual search string with Boolean operators. Please provide an example of the full search strategy for one database such as PubMed. | We have now added a full example of the search strategy for PubMed in the Methods section (Search strategy subsection). The example includes all Boolean operators (AND, OR), Mesh terms(aged), truncation (digni*), and the date restriction (from 1966 01 January to 31 August 2025). | Line179-188 | Minor |
| **Eight.** | Author roles are unclear. You mentioned a researcher, an assistant graduate student, and a non-nursing assistant. Their qualifications are not described. Please clarify who these individuals are and their level of training. | We have described the roles of all the team members and described their level of training. | Line189-204 | Major |
| **Nine.** | JBI cutoff scores need justification. You set 80% for high quality and 50% for low quality. Please provide a reference or justification for these specific cutoff scores. | We cited the reference and described the justification for the cutoff score. | Line259-267 | Minor |
| **Ten.** | Low quality studies decision is vague. You stated low quality studies will be "reviewed and discussed" with a decision to retain or exclude. This is subjective. Please specify clear criteria for when a low quality study will be retained versus excluded. | According to the JBI manual of systematic review,we added an exclusion criteria. | Line267-278 | Major |
| **Discussion:** This section is well written. However I have several comments. | | | | |
| **One.** | You stated that themes related to basic safety needs and physical care needs are anticipated within inherent dignity. Please clarify whether these needs are based on a specific theoretical framework such as Maslow or a nursing theory. If not, consider adding a brief reference. | We agree with your comments and have added Maslow’s Hierarchy of Needs Theory to underpin the themes related to basic safety and physical care needs within inherent dignity. | Line363-366 | Minor |
| **Two.** | You stated that personal factors such as functional status, organizational factors such as ethical care environment, and sociocultural factors such as collectivism or ageism may influence dignity. This is good but please provide at least one reference for each category of factors to strengthen your argument. | Follow your comments we have added the reference for each factor to enhance the credibility. | Line367-373 | Minor |
| **Three.** | You compared your expected findings to previous reviews and cited references 37, 38, and 39. However you did not specify what these previous reviews found and how your review will differ. Please add one or two sentences explaining the specific gaps in those previous reviews that your review will address. | Thank you for this constructive suggestion. We agree that the original text lacked specificity regarding the limitations of the cited reviews. We have revised the manuscript accordingly by adding two sentences to clarify the gaps in the previous reviews and how our review will address them. | Line 382-386 | Minor |
| **Four.** | You mentioned challenges in synthesizing themes that accurately represent original findings. This is a valid concern. However you did not mention how you will handle conflicting findings across studies. Please add a brief statement about how disagreements in synthesized themes will be resolved. | We agree that addressing how conflicting findings across studies will be handled is essential for methodological transparency. In response, we have added a sentence to the clarify that conflicting findings. | Line397-402 | Minor |
| **Five.** | You stated that future research should incorporate multiple demographic perspectives. Please specify which demographics you mean, for example age subgroups, gender, or cultural backgrounds. | Follow your advice, we add the specific information in the demographics. | Line 403-412 | Major |
| **Limitations:** This section is honest and well written. However I have several comments. | | | | |
| **One.** | You acknowledged language bias and the use of DeepL translation. This is good. However you did not mention the possibility that some non-English and non-Chinese studies might be missed entirely if they are not indexed in your selected databases. Please add this. | Follow your comments, we added this item in our limitation. | Line 418-419 | Minor |
| **Two** | You acknowledged exclusion of grey literature and the risk of publication bias. This is good. However you did not mention the possibility that excluding theses and dissertations may miss emerging research from early career researchers. Please consider adding this point. | We agree that it is an important limitation that warrants explicit acknowledgement. We have revised the sentence accordingly to include this point. | Line423-425 | Minor |
| **Three** | You stated that findings may not be fully transferable to other cultural contexts given Chinese cultural values. This is a valid limitation. However you did not mention that findings may also not be transferable to other Asian cultures such as Japanese or Korean which have different aging and long-term care systems. Please add this. | We agree that specifying the limitation of transferability even within Asian cultural contexts strengthens the rigor of our discussion. We have added a sentence acknowledging that the findings may not be directly applicable to other Asian contexts. | Line 429-432 | Minor |
| **Four.** | You did not mention the risk of reviewer bias in coding and theme development even though two reviewers will extract data independently. Please add this as a limitation because no matter how independent the reviewers are, their interpretations may still be influenced by their own cultural and professional backgrounds. | We agree that reviewer bias and we added this point in the limitation section. | Line 432-439 | Minor |
| **Five.** | You did not mention that the search will be conducted at a single point in time and any studies published after August 2025 will not be included. This is a standard limitation for any systematic review. Please add it. | This is an important point in the systematic review. Follow this comment, we add this standard limitation in the limitation section. | Line 439-441 | Minor |
| **Conclusions:** This section is well written. However I have several comments. | | | | |
| **One.** | You stated that this review aims to synthesize qualitative evidence including Chinese literature. This is already stated in your aim. Consider making your conclusion more impactful by stating the expected clinical or policy implications explicitly rather than repeating the aim. | In the revised manuscript, we have removed the restatement of the aim and replaced it with explicit clinical and policy implications drawn from our findings. | Line448-451  Line460-467 | Major |
| **Two.** | You recommended future research to explore dignity from perspectives of nursing staff, family members, administrators, and policymakers. This is good. However you did not mention the perspective of the older adults themselves which you already included. Please rephrase to avoid confusion. | We would like to clarify that older adults' perspectives are already included in the future research recommendation: *"not only from older adults' perspectives, but also from the perspectives of nursing staff, family members, administrators, and policymakers."* We have carefully reviewed the sentence and confirmed that older adults are explicitly mentioned. We hope this clarifies any confusion. | Line468 | Minor |
| **Reference** | | | | |
| **One.** | Reference number 1 is from 2026. You are writing this systematic review protocol in 2024 or 2025. A 2026 publication cannot exist at the time you are writing your protocol. Please check the year. If this is a future publication, you cannot cite it. If the year is a typo, please correct it to the actual publication year. | We have revised inappropriate reference. | Ref 2:Line 514-516 | Minor |
| **Two.** | Reference number 25 is DeepL Translator 2026. Same issue. The year 2026 has not arrived yet. Please correct to the actual version year or the year you accessed the tool. | We have revised inappropriate reference. | Ref 30 Line 605-606 | Minor |
| **Three.** | Reference number 3 is WHO 2015. This is a valid reference. However the WHO has more recent reports on ageing and health such as the Decade of Healthy Ageing 2021-2030 reports. Consider adding more recent WHO sources. | We agree that more recent WHO sources would strengthen the evidence base of this manuscript. We cited the new similar reference that described the same meaning of the sentences. | Ref3 Line517-519 | Major |
| **Four** | Reference number 16 is the Universal Declaration of Human Rights 1948. This is correct. However you are citing this as a source for inherent dignity. Consider also citing more recent commentaries on human dignity and ageing. | We have replaced the outdated reference and added the new references. | Ref 16,17.  Line559-564 | Major |
| **Five.** | Reference number 20 is PRISMA-P 2015. This is correct for your protocol. However ensure you also cite PRISMA 2020 for the review itself. You mentioned PRISMA 2020 in your methods but I do not see it cited separately. Please add reference 21 as PRISMA 2020 statement. You have it but check the citation format. | Thank you for this comment. We confirm that both PRISMA-P 2015 and PRISMA 2020 are cited in the methods section and as your comment six in methods section. Specifically, we stated that the protocol is reported in accordance with the PRISMA-P 2015 statement, and that the completed review will be reported in accordance with the PRISMA 2020 guidelines. Both references are included in the reference list. | Line 119-121 | Minor |
| **Six.** | Reference number 31 is Hannes et al 2010. This is a comparative analysis of appraisal tools. However this study is 14 years old. Consider adding more recent evidence comparing JBI with other tools. | Thank the reviewers for this valuable suggestion. We compared with the CASP and JBI tools and found that the emphasis between which have different emphasis. The revised text now emphasizes the JBI tool's focus on congruity across the research process — from philosophical perspective through to methodology and analytical approach — as the primary justification for its selection, rather than relying solely on a comparative argument based on Hannes et al. (2010). | Reference 35 | Major |
| **Seven.** | References 32 and 33 are about AMSTAR. You are using JBI, not AMSTAR. Why are you citing AMSTAR validation studies? These references seem irrelevant to your JBI-based quality appraisal. Please either justify why these are included or remove them. | We remove the unrelated references 32 and 33. | Removed | Major |
| **Eight.** | Reference number 37 is Wachholz and Giacomin 2022. This is a commentary piece from F1000Research. Please verify that this is a peer-reviewed source appropriate for your discussion. | Thank you for this important observation. We acknowledge that Wachholz and Giacomin (2022), previously cited as reference 37, is a commentary piece and may not constitute the most robust form of peer-reviewed evidence for this claim. We have therefore revised this section and replaced the original reference with more appropriate peer-reviewed sources. | Reference 52-53-54-55  Line672-684 | major |
| **Nine.** | Reference number 38 is Šaňáková and Čáp 2019. This is a qualitative literature review. It is appropriate. No issue. | Thank you for your confirmation. We have checked this reference and no changes have been made to this reference. |  | minor |
| **Ten.** | Reference number 39 is Hall et al 2014. This is ten years old at the time of your review. Consider adding more recent studies on dignity from multiple perspectives. | It is an older reference, and we use latest reference to replace it. |  | Major |
