# Supplementary material for: The perception and experience of dignity in the care of older adults in nursing homes: A Meta-aggregation protocol
Source: PLoS One. 2026 Jul 21;21(7):e0351774. doi: 10.1371/journal.pone.0351774 (PMC13387536; doi:10.1371/journal.pone.0351774)
Supplement: Attachment — Submitted filename: Response_to_Reviewers_auresp_3.docx [file pone.0351774.s013.docx]

Dear Editor and Reviewers,

Thank you for your valuable time and constructive comments on our manuscript. We have carefully addressed all the concerns raised and revised the manuscript accordingly. Below, we provide a point-by-point response to each comment. All changes in the revised manuscript are highlighted in track changes.

We hope that our revisions are satisfactory and look forward to your favourable consideration.

Sincerely,

[Chunxiang Li]

| **Location** | **Reviewer comments** | **Response** | **Location in MS** | **Type(Major or Minor)** |
| --- | --- | --- | --- | --- |
| **ABSTRACT** |  |  |  |  |
| 1 | Shorten the aim. Example: "This systematic review aims to conceptualize the characteristics of dignity in nursing home care for older adults, with attention to Chinese cultural perspectives." | We have revised the aim concisely. | Line13-16 | Minor |
| 2 | Reword reviewer roles for clarity: "Three reviewers will screen titles and abstracts. Two reviewers will then independently select studies and extract data using predesigned forms. Any discrepancies will be documented and resolved by a third reviewer until consensus is reached." | We have revised the manuscript to clarify the roles of the reviewers in the screening process. | Line 26-30 | Minor |
| 3 | Remove redundant "assistance... assist" – use "a third reviewer will resolve discrepancies". | We have removed the redundant words . | Line 31-32 | Minor |
| 4 | Change "will be clearly claimed" to "will be documented" | We have changed to the appropriate words. |  | Minor |
| **Introduction** | The introduction is well‑written and covers the necessary background. No revisions needed. | No need to revise. | | |
| **Methods Section:** | The overall structure is clear and follows standard systematic review protocol guidelines. However, a few minor revisions are needed: |  |  |  |
| 1 | Correct typo: change "system review" to "systematic review". | We have revised the wrong words. | Line153 | Minor |
| 2 | Minor language fix: change "older adults live in nursing homes" to "older adults living in nursing homes". | We have revised the wrong words. | Line 135 | Minor |
| 3 | Grey literature justification is good but could be shortened (optional). | Noted. We have merged the two sentences and revised them concisely. | Line157-162 | Minor |
| (1) | Typo in Table title – "Seach Strategy" should be "Search Strategy" (Table 2). | Revised the wrong word. | Line166 | Minor |
| (2) | Redundant sentence – In the first paragraph, you have: "The search covered the period from 1966 to 31 August 2025" – this is already stated twice (once with database inception, once at the end). Remove the second mention. | Removed the redundant sentence. | Line178 | Minor |
| (3) | Reviewer roles – Clarify who does what. You describe 3 reviewers with different backgrounds, but then state that study selection and critical appraisal are performed by two reviewers (primary researcher + nursing graduate student). The non‑nursing assistant does initial screening only. This is fine, but ensure consistency in the description. | We have revised the manuscript to clarify the roles of each reviewer and ensure consistency throughout the description. | Line 207-242 | Minor |
| (4) | Typo – "assitantant" should be "assistant" (in reviewer team description). | Revised the wrong word. | Line 191 | Minor |
| (5) | Minor language – "will be jointly responsible" → "will be jointly responsible" (remove extra "s" in "jointly"? already correct). "cannot be available" → "are not available". | Revised the wrong word. | Line 194-195,236-237 | Minor |
| (6) | Low‑quality study exclusion – Your plan is good (retain by default, exclude only if critical flaw). However, the sentence is long. Consider shortening for clarity. | We shorten the sentences concisely following your suggestions. | Line 265-279 | Minor |
| (7) | Missing PROSPERO registration number – As noted earlier, recommended for transparency. | We added the registration number (CRD420251020581) for transparency. | Line299-300 | Minor |
| (8) | Timeline typo – "Januray" should be "January" , "Februray" should be "February" . | Revised the wrong word. | Line353 | Minor |
| **The discussion section** is well‑written, comprehensive, and appropriately linked to the theoretical framework. No major revisions are needed. | | No need to revise. | | |
| **These final sections** are well‑written and require only a few minor correction: | |  |  |  |
| 1 | Change "the study will exclude" to "this review will exclude" . | We have revised the expressions. | Line 423 | Minor |
| 2 | Simplify "there lies a potential interpretive bias" to "interpretive bias is a potential limitation" . | Noted. We have simplified the expression as suggested. | Line 427-428 | Minor |
| 3 | List of Abbreviations: Correct "Phenomenonon of interest" to "Phenomenon of interest" . | Noted. We have corrected the wrong word. | Line463 | Minor |
| 4 | Support Information  Correct "S2 Table. Table 2 Search Strategy" to "S2 Table. Search Strategy" (remove duplicate "Table 2")。 | We have removed the duplicate contents. | Line 494 | Minor |
| **The reference list** is comprehensive. A few minor corrections are needed: | |  |  |  |
| **1** | Reference 15: Incomplete DOI. Please correct or remove it. | Revised the correct Doi as 10.1177/0969733020961822. | Line547 | Minor |
| 2 | Reference 29: Empty DOI placeholder (https://doi.org/https://doi.org/). Please remove or provide the correct DOI. | Noted. This reference is a book and does not have a DOI. It has been removed accordingly. | Line 589 | Minor |
