# Supplementary material for: The perception and experience of dignity in the care of older adults in nursing homes: A Meta-aggregation protocol
Source: PLoS One. 2026 Jul 21;21(7):e0351774. doi: 10.1371/journal.pone.0351774 (PMC13387536; doi:10.1371/journal.pone.0351774)
Supplement: Peer review history

# Editor report: Decision Letter 0

**DOI:** 10.1371/journal.pone.0351774.r001

**Authors:** Samar Sobhi

13 Feb 2026

PONE-D-25-46837

The perception and experience of dignity in the care of older adults in nursing homes: A Meta-aggregation protocol

PLOS One

Dear Dr. Li,

Thank you for submitting your manuscript to PLOS ONE. After careful consideration, we feel that it has merit but does not fully meet PLOS ONE’s publication criteria as it currently stands. Therefore, we invite you to submit a revised version of the manuscript that addresses the points raised during the review process.

Please submit your revised manuscript by Mar 30 2026 11:59PM. If you will need more time than this to complete your revisions, please reply to this message or contact the journal office at plosone@plos.org. When you’re ready to submit your revision, log on to https://www.editorialmanager.com/pone/ and select the 'Submissions Needing Revision' folder to locate your manuscript file.

Please include the following items when submitting your revised manuscript:

- A letter that responds to each point raised by the academic editor and reviewer(s). You should upload this letter as a separate file labeled 'Response to Reviewers'.
- A marked-up copy of your manuscript that highlights changes made to the original version. You should upload this as a separate file labeled 'Revised Manuscript with Track Changes'.
- An unmarked version of your revised paper without tracked changes. You should upload this as a separate file labeled 'Manuscript'.

If you would like to make changes to your financial disclosure, please include your updated statement in your cover letter. Guidelines for resubmitting your figure files are available below the reviewer comments at the end of this letter.

If applicable, we recommend that you deposit your laboratory protocols in protocols.io to enhance the reproducibility of your results. Protocols.io assigns your protocol its own identifier (DOI) so that it can be cited independently in the future. For instructions see: https://journals.plos.org/plosone/s/submission-guidelines#loc-laboratory-protocols. Additionally, PLOS ONE offers an option for publishing peer-reviewed Lab Protocol articles, which describe protocols hosted on protocols.io. Read more information on sharing protocols at https://plos.org/protocols?utm_medium=editorial-email&utm_source=authorletters&utm_campaign=protocols.

We look forward to receiving your revised manuscript.

Kind regards,

Samar Sobhi

Guest Editor

PLOS One

**Journal Requirements:**

When submitting your revision, we need you to address these additional requirements.

1. Please ensure that your manuscript meets PLOS ONE's style requirements, including those for file naming. The PLOS ONE style templates can be found at

https://journals.plos.org/plosone/s/file?id=wjVg/PLOSOne_formatting_sample_main_body.pdf and

https://journals.plos.org/plosone/s/file?id=ba62/PLOSOne_formatting_sample_title_authors_affiliations.pdf

2. Thank you for stating the following in the Acknowledgments Section of your manuscript:

“We gratefully acknowledge the support provided by Henan University of Science and Technology for this research. The first and third authors are supported by the 2024 National Student Innovation and Entrepreneurship Training Program at Henan University of Science and Technology (Grant No. 202410464059). This work is also funded by the Experimental Technology Development Fund Program of Henan University of Science and Technology for the academic year 2024–2025 (No. SY2425057). The funders had no role in the design, execution, or interpretation of this study.”

We note that you have provided funding information that is not currently declared in your Funding Statement. However, funding information should not appear in the Acknowledgments section or other areas of your manuscript. We will only publish funding information present in the Funding Statement section of the online submission form.

Please remove any funding-related text from the manuscript and let us know how you would like to update your Funding Statement. Currently, your Funding Statement reads as follows:

“The author(s) received no specific funding for this work.”

Please include your amended statements within your cover letter; we will change the online submission form on your behalf.

3. If the reviewer comments include a recommendation to cite specific previously published works, please review and evaluate these publications to determine whether they are relevant and should be cited. There is no requirement to cite these works unless the editor has indicated otherwise.

**Additional Editor Comments:**

1-ABSTRACT: 5 mandatory required

1. BACKGROUND: State gap clearly.

2. AIM :Rewrite, one clear sentence.

3.METHODS :Add missing items. "Quality will be assessed using JBI. Meta-aggregation will be used for synthesis."

4. EXPECTED OUTCOMES: Be specific include key themes, domains, and cultural factors influencing dignity.

1. LANGUAGE: Professional language editing required.

2- The keywords in the abstract have been revised by replacing “older people” with “older adults” to ensure consistency with the title.

3- In introduction:

1. Restructure the introduction using a clear problem → gap → aim → research question framework. The current version is fragmented and repetitive.

2. State the research question explicitly. A systematic review protocol must clearly define what it seeks to answer (e.g., “What are the perceptions and experiences of dignity among older adults in nursing homes?”).

3. Align the introduction with the abstract. The abstract emphasizes Chinese cultural context, yet the introduction makes no mention of it. This inconsistency must be resolved.

4. Undergo professional language editing. The manuscript contains frequent grammatical errors, spelling mistakes, and awkward phrasing that obscure meaning and reduce credibility.

5. Specify the theoretical framework. Explicitly state which dignity model (e.g., Jacobson, Kitwood) will guide the synthesis and interpretation of findings.

6. Justify the need for a systematic review. Explain why this review is necessary now, what previous reviews have missed, and how this review will contribute new knowledge.

4-methodology is clear, appropriate, and well-structured Just 4 Issues to Fix Before Resubmission:

1 Research Questions – Poorly framed, grammar errors, no PEO structure Rewrite using PEO framework, fix singular/plural, clarify Chinese context

2 Grey Literature – Too vague ("all qualitative documents found") Specify sources (ProQuest, OpenGrey, Google Scholar) and types (theses, conference proceedings)

3 Non-English Translation – Unclear process, grammar error ("will be translate") Clarify translation method (e.g., DeepL) and add bilingual verification

4 Language – Pervasive spelling/grammar errors throughout Professional language editing is mandatory.

5- Discussion section needs 4 fixes:

1. Language – Errors are everywhere ("angles", "culture care", "literatures"). Professional editing is mandatory.

2. Tone – You have no results yet. Do not say "will". Use "expected to" or "may" .

3. Limitations – You forgot language bias, publication bias, and cultural generalizability. Add them.

4. Missing – Add your PROSPERO number, how you will share findings, and a PPI statement.

6- Conclusion needs 4 fixes:

1. Language – "include" → "including", "will be synthesize" → "will be synthesized", "system review" → "systematic review", "nursing home" → "nursing homes".

2. Tone – You have no results yet. Do not say "will" . Say "aims to" or "is expected to" .

3. Overclaiming – One review cannot produce "guidelines" . Say "inform strategies" instead.

4. Missing – Add Chinese cultural context and a brief future research sentence.

funding statement and supplementary files are excellent. However, 4 fixes are required:

1. Author Contributions – Too vague ("screens literature"). Use CRediT taxonomy (Conceptualization, Methodology, Investigation, Writing, Supervision, etc.). Also identify the corresponding author.

2. Missing statements – Add Conflict of Interest ("The authors declare no conflicts") and Data Availability ("No new data were generated").

3. Abbreviations – Fix CASP (Critical Appraisal Skills Programme), PICo capitalization, and PROSPERO colon.

4. Supplementary files – Fix "S 3File" → "S3 File", and correct "Seach Strategy" spelling.

7-Reference list:5 fixes are required:

1. Pick ONE style – Vancouver or APA. Do not mix them.

2. Add DOIs – almost all are missing. This is required in 2025.

3. Fix incomplete references – Ref 8 ("no-no") is unacceptable. Provide full page numbers. Complete Ref 5, 16, 18.

4. Check in-text citations – Refs 12,13,14,16,17,21,22 may not be cited in your manuscript. Add or remove.

5. Correct typos – Ref 1 (Oxford), Ref 2 (parentheses), Ref 23 (journal name capitalization).

[Note: HTML markup is below. Please do not edit.]

[NOTE: If reviewer comments were submitted as an attachment file, they will be attached to this email and accessible via the submission site. Please log into your account, locate the manuscript record, and check for the action link "View Attachments". If this link does not appear, there are no attachment files.]

To ensure your figures meet our technical requirements, please review our figure guidelines: https://journals.plos.org/plosone/s/figures

You may also use PLOS’s free figure tool, NAAS, to help you prepare publication quality figures: https://journals.plos.org/plosone/s/figures#loc-tools-for-figure-preparation.

NAAS will assess whether your figures meet our technical requirements by comparing each figure against our figure specifications.

# Author comment: Author response to Decision Letter 1

**DOI:** 10.1371/journal.pone.0351774.r002

1 Apr 2026

Dear Editor and Reviewers,

Thank you for your valuable time and constructive comments on our manuscript. We have carefully addressed all the concerns raised and revised the manuscript accordingly. Below, we provide a point-by-point response to each comment. All changes in the revised manuscript are highlighted in track changes.

We hope that our revisions are satisfactory and look forward to your favourable consideration.

Sincerely,

[Chunxiang Li]

Reviewer comments Response Location in MS Type(Major or Minor)

1 Resubmitting your figure files. S2 Fig, S3 Fig Support information Major

2 File naming S1 Table, S2 Table, S1,S2 S3 Fig, S1,S2 S3 File Support information Major

3 Acknowledgments Section Have added in the cover letter and deleted in the acknowledgements of manuscript. Acknowledgments Section Major

4 Recommendation to cite specific previously published works No No

Reviewer comments

1-ABSTRACT: 5 mandatory required All five required items have been addressed in the revised abstract. Line10-34 Major

�1�BACKGROUND: State gap clearly. Clarified the research gap in the Background. Line11-12 Major

�2�AIM :Rewrite, one clear sentence. Rewritten the Aim as one clear sentence. Line 13-16 Major

�3�METHODS :Add missing items. "Quality will be assessed using JBI. Meta-aggregation will be used for synthesis." Added the missing quality assessment method. Lin25-27 Major

�4�EXPECTED OUTCOMES: Be specific include key themes, domains, and cultural factors influencing dignity. Specific in the themes, domains and factors in expected outcomes. Line28-34 Major

2. LANGUAGE: Professional language editing required. The language has been improved with the assistance of a PhD colleague who has extensive publication experience. Whole manuscript Major

3- The keywords in the abstract have been revised by replacing “older people” with “older adults” to ensure consistency with the title. Thank you for this revision. Whole manuscript and keywords in the system Minor

4- Introduction

(1) Restructure the introduction using a clear problem → gap → aim → research question framework. The current version is fragmented and repetitive. Problem: line 39-60, gap: Line 58-65, aim: Line83-90. Research questions: Line 92-97

Line 38-97 Major

(2) State the research question explicitly. A systematic review protocol must clearly define what it seeks to answer (e.g., “What are the perceptions and experiences of dignity among older adults in nursing homes?”). There are two specific research questions. Line93- 97 Major

(3) Align the introduction with the abstract. The abstract emphasizes Chinese cultural context, yet the introduction makes no mention of it. This inconsistency must be resolved. Added a paragraph in the introduction to explicitly discuss the Chinese cultural context (e.g., collectivism, and their influence on dignity in care for older adults). This revision ensures consistency with the abstract. Please see [Section: Introduction, Line 39-57, line75-81]. Line 39-57,

line 75-81 Major

(4) Undergo professional language editing. The manuscript contains frequent grammatical errors, spelling mistakes, and awkward phrasing that obscure meaning and reduce credibility. (1)Grammarly was used to correct grammatical and spelling errors; (2) A PhD colleague with extensive publication experience copy-edited the manuscript for clarity and readability. Whole manuscript Major

(5) Specify the theoretical framework. Explicitly state which dignity model (e.g., Jacobson, Kitwood) will guide the synthesis and interpretation of findings. (1) Jacobson dignity theoretical framework help to identify the factors and domains of dignity.(2) The function of this theory has been interpreted in introduction section [Line 66-74, line85-88]. Line66-74, line85-88 Major

(6) Justify the need for a systematic review. Explain why this review is necessary now, what previous reviews have missed, and how this review will contribute new knowledge. Why now: Although dignity in care for older adults in nursing homes has received increasing research attention, the evidence has not been systematically synthesized to inform practice . Line 61-65.

What previous reviews have missed: no systematic review has synthesized qualitative evidence incorporating the Chinese cultural context. Line 76-81.

New knowledge contribution: generating evidence to inform dignified care strategies for Chinese nursing home settings. Line 82-90. Line 61-65. Line 76-81.Line 92-90. Major

5-methodology is clear, appropriate, and well-structured Just 4 Issues to Fix Before Resubmission:

(1) Research Questions – Poorly framed, grammar errors, no PEO structure Rewrite using PEO framework, fix singular/plural, clarify Chinese context 1. What are the perceptions and experiences of dignity (O) among older adults (P) in nursing homes across Western and Chinese cultural contexts (E)?

2. What personal, organizational, and sociocultural factors (E) influence dignity (O) among older adults (P) in nursing homes across Western and Chinese cultural contexts?

Line 93-97 Major

(2) Grey Literature – Too vague ("all qualitative documents found") Specify sources (ProQuest, OpenGrey, Google Scholar) and types (theses, conference proceedings) Specified the grey literature sources and types in the Exclusion criteria section. Line142-144 minor

(3) Non-English Translation – Unclear process, grammar error ("will be translate") Clarify translation method (e.g., DeepL) and add bilingual verification We have corrected the grammar error (“will be translate” → “will be translated”). We have also clarified that non-English articles will be translated using DeepL, followed by bilingual verification by a a bilingual expert or postgraduate student proficient in the source language. Line186-189 Major

(4) Language – Pervasive spelling/grammar errors throughout Professional language editing is mandatory. (1)Grammarly was used to correct grammatical and spelling errors; (2) A PhD colleague with extensive publication experience copy-edited the manuscript for clarity and readability. Whole manuscript Major

5- Discussion section needs 4 fixes:

(1)Language – Errors are everywhere ("angles", "culture care", "literatures"). Professional editing is mandatory.

(1)Grammarly was used to correct grammatical and spelling errors; (2) A PhD colleague with extensive publication experience copy-edited the manuscript for clarity and readability. Whole manuscript Major

(2) Tone – You have no results yet. Do not say "will". Use "expected to" or "may" . Revised the “will” to “expected to” Line292 Minor

(3) Limitations – You forgot language bias, publication bias, and cultural generalizability. Add them. (1) Language bias: machine translation may not fully capture the nuanced meanings of dignity. Line324-327

(2) Publication bias by potentially overlooking relevant studies not indexed in peer-reviewed databases. Line 327-330.

(3) Not be fully transferable to other cultural contexts.Line 330-333. Line324-333 Major

(4) Missing – Add your PROSPERO number, how you will share findings, and a PPI statement. Add the PROSPERO number: CRD420251020581. Line 334-338 Major

6- Conclusion needs 4 fixes:

(1) Language – "include" → "including", "will be synthesize" → "will be synthesized", "system review" → "systematic review", "nursing home" → "nursing homes". All four have been corrected. Additionally, we have proofread the entire manuscript to ensure no similar errors remain. Whole manuscript Major

(2) Tone – You have no results yet. Do not say "will" . Say "aims to" or "is expected to" . Replace future-tense predictions. Line345 Major

(3)Overclaiming – One review cannot produce "guidelines" . Say "inform strategies" instead. Replace the term “guidelines” with more accurate phrasing “inform strategies” Line345-347 Major

(4) Missing – Add Chinese cultural context and a brief future research sentence. Add the Chinese cultural context and future research. Line347-353 Major

Funding statement and supplementary files are excellent. However, 4 fixes are required:

(1) Author Contributions – Too vague ("screens literature"). Use CRediT taxonomy (Conceptualization, Methodology, Investigation, Writing, Supervision, etc.). Also identify the corresponding author. Use CRediT taxonomy to describe each author’s contribution. And the corresponding author. Line 362-367 Major

(2)Missing statements – Add Conflict of Interest ("The authors declare no conflicts") and Data Availability ("No new data were generated"). Add Conflict of Interest and Data Availability. Line 369-370 Major

(3)Abbreviations – Fix CASP (Critical Appraisal Skills Programme), PICo capitalization, and PROSPERO colon. Abbreviation section has been revised. Line355-358 Minor

(4) Supplementary files – Fix "S 3File" → "S3 File", and correct "Seach Strategy" spelling. Supplementary files have been revised following the requirements of PLOS ONE. Line371-391 Major

7-Reference list:5 fixes are required:

1. Pick ONE style – Vancouver or APA. Do not mix them. All references consistently follow Vancouver (Plos One’s　format　 https://journals.plos.org/plosone/article?id=10.1371/journal.pone.0031009#references) throughout the manuscript. Line 392-516 Major

2. Add DOIs – almost all are missing. This is required in 2025. Add DOIs for all references where DOIs are available. Line 392-517 Major

3. Fix incomplete references – Ref 8 ("no-no") is unacceptable. Provide full page numbers. Complete Ref 5, 16, 18. Ref 8 (previously “no-no”) has been removed following revisions to the Introduction section.

Page numbers have been added where required.

Ref 5 has been corrected to: Ref 18:Gallagher A (2004) Dignity and respect for dignity - Two key health professional values: Implications for nursing practice. Nursing Ethics 11:587-599.https://doi.org/10.1191/0969733004ne744oa.

Ref16 has been corrected to: Ref 27: Bettany-Saltikov J, McSherry R(2024) How to do a Systematic Literature Review in Nursing: A Step-by-Step Guide (3rd ed.): Open University Press.

Ref 18 has been corrected to: Ref 29: Aromataris E, Lockwood C, Porritt K, Pilla B, Jordan Z (2024) JBI manual for evidence synthesis.https://doi.org/10.46658/JBIMES-24-01.

Ref 5: Line 445-447

Ref 16: Line 472-47

Ref 18: Line477-478 Major

4. Check in-text citations – Refs 12,13,14,16,17,21,22 may not be cited in your manuscript. Add or remove. Refs 12, 13, 14, 16, 17, 21, 22 have been reviewed. Uncited references have been removed; newly cited references have been added accordingly.

Line 392-517 Major

5. Correct typos – Ref 1 (Oxford), Ref 2 (parentheses), Ref 23 (journal name capitalization). Ref 1 has been removed following revisions to the Introduction section.

Ref 2 has been revised as:

Ref 2:United Nations (2017) World Population Ageing 2017: Highlights. https://www.un.org/development/desa/pd/sites/www.un.org.development.desa.pd/

files/files/documents/2020/May/un_2017_worldpopulationageing_report.pdf

Ref 23 has been revised as: Ref 34: Thomas J, Harden A (2008) Methods for the thematic synthesis of qualitative research in systematic reviews. BMC medical research methodology 8:1-10.https://doi.org/10.1186/1471-2288-8-45.

Ref 2: Line 396-398

Ref 23: Line 493-495 Major

Attachment Submitted filename: Response to Reviewers.docx

# Editor report: Decision Letter 1

**DOI:** 10.1371/journal.pone.0351774.r003

**Authors:** Samar Sobhi

12 Apr 2026

PONE-D-25-46837R1

The perception and experience of dignity in the care of older adults in nursing homes: A Meta-aggregation protocol

PLOS One

Dear Dr. Li,

Thank you for submitting your manuscript to PLOS ONE. After careful consideration, we feel that it has merit but does not fully meet PLOS ONE’s publication criteria as it currently stands. Therefore, we invite you to submit a revised version of the manuscript that addresses the points raised during the review process.

Please submit your revised manuscript by May 27 2026 11:59PM. If you will need more time than this to complete your revisions, please reply to this message or contact the journal office at plosone@plos.org. When you're ready to submit your revision, log on to https://www.editorialmanager.com/pone/ and select the 'Submissions Needing Revision' folder to locate your manuscript file.

Please include the following items when submitting your revised manuscript:

- A letter that responds to each point raised by the academic editor and reviewer(s). You should upload this letter as a separate file labeled 'Response to Reviewers'.
- A marked-up copy of your manuscript that highlights changes made to the original version. You should upload this as a separate file labeled 'Revised Manuscript with Track Changes'.
- An unmarked version of your revised paper without tracked changes. You should upload this as a separate file labeled 'Manuscript'.

If you would like to make changes to your financial disclosure, please include your updated statement in your cover letter. Guidelines for resubmitting your figure files are available below the reviewer comments at the end of this letter.

If applicable, we recommend that you deposit your laboratory protocols in protocols.io to enhance the reproducibility of your results. Protocols.io assigns your protocol its own identifier (DOI) so that it can be cited independently in the future. For instructions see: https://journals.plos.org/plosone/s/submission-guidelines#loc-laboratory-protocols. Additionally, PLOS ONE offers an option for publishing peer-reviewed Lab Protocol articles, which describe protocols hosted on protocols.io. Read more information on sharing protocols at https://plos.org/protocols?utm_medium=editorial-email&utm_source=authorletters&utm_campaign=protocols.

As the corresponding author, your ORCID iD is verified in the submission system and will appear in the published article. PLOS supports the use of ORCID, and we encourage all coauthors to register for an ORCID iD and use it as well. Please encourage your coauthors to verify their ORCID iD within the submission system before final acceptance, as unverified ORCID iDs will not appear in the published article. *Only*  the individual author can complete the verification step; PLOS staff *cannot*  verify ORCID iDs on behalf of authors.

We look forward to receiving your revised manuscript.

Kind regards,

Samar Sobhi

Guest Editor

PLOS One

Journal Requirements:

If the reviewer comments include a recommendation to cite specific previously published works, please review and evaluate these publications to determine whether they are relevant and should be cited. There is no requirement to cite these works unless the editor has indicated otherwise.

Please review your reference list to ensure that it is complete and correct. If you have cited papers that have been retracted, please include the rationale for doing so in the manuscript text, or remove these references and replace them with relevant current references. Any changes to the reference list should be mentioned in the rebuttal letter that accompanies your revised manuscript. If you need to cite a retracted article, indicate the article’s retracted status in the References list and also include a citation and full reference for the retraction notice.

Additional Editor Comments:

Your abstract is well written but three essential elements are missing. Please add the following to your abstract before resubmission.

One. Please specify the search timeframe. You need to state the period of the search, for example from inception to 2024 or from 2000 to 2024.

Two. Please specify your inclusion and exclusion criteria. You need to state the language criteria such as English and Chinese only. You also need to state whether grey literature such as conference proceedings and dissertations will be included or excluded.

Three. Please add transparency and reproducibility elements.

Your introduction is well written and has several strengths. The problem is clearly stated, the aim is specific, the justification for the review is clear, the theoretical framework of Jacobson and Høy is properly introduced, the Chinese cultural context is explicitly addressed, and the gap in literature is well identified.

However I have the following comments for improvement:

One. Please check your in-text citations. Some numbers appear without corresponding references. Ensure every citation number has a full reference in your reference list.

Two. Your first paragraph is very long. Consider breaking it into two shorter paragraphs for better readability.

Three. You wrote "hybrid analytical approach" in the last paragraph but you did not explain what this means. Please clarify this term or remove it if not necessary.

Four. You stated that this review will use meta-aggregation and then examine findings through Jacobson's framework. Please clarify whether the framework will guide the synthesis or will be applied after the synthesis. needs to be explicit.

methods section is strong and well developed. The ten issues identified need to be corrected.

One, date inconsistency. You wrote "31st September 2025" as your search completion date. September has 30 days, not 31. Please correct to 30th September 2025.

Two, year inconsistency in timeline. In your timeline you wrote October 1-October 31, 2025 and November 1-November 30, 2025 but your search completion date is August 31, 2025. These dates do not match. Please revise your timeline to be consistent with your search completion date.

Three, missing justification for excluding grey literature. You excluded theses, dissertations, and non-peer-reviewed materials. This is a major limitation that may introduce publication bias. Please provide a clear rationale for why grey literature is excluded.

Four, missing explanation for excluding older adults with Alzheimer's disease. You excluded older adults with severe Alzheimer's or psychological disease. Please explain why this population is excluded as their dignity is also an important concern.

Five, translation method issue. You stated you will use DeepL for non-English and non-Chinese literature. Machine translation may miss nuanced meanings of dignity. Please add that you will have a bilingual expert verify all translated content.

Six, PRISMA version inconsistency. You cited PRISMA 2015 for the protocol and PRISMA 2020 for the review. Please use PRISMA 2020 for both or clarify why different versions are used.

Seven, missing sample of search strategy. You provided Table 2 but did not show the actual search string with Boolean operators. Please provide an example of the full search strategy for one database such as PubMed.

Eight, author roles are unclear. You mentioned a researcher, an assistant graduate student, and a non-nursing assistant. Their qualifications are not described. Please clarify who these individuals are and their level of training.

Nine, JBI cutoff scores need justification. You set 80% for high quality and 50% for low quality. Please provide a reference or justification for these specific cutoff scores.

Ten, low quality studies decision is vague. You stated low quality studies will be "reviewed and discussed" with a decision to retain or exclude. This is subjective. Please specify clear criteria for when a low quality study will be retained versus excluded.

Discussion

This section is well written. However I have several comments.

One, you stated that themes related to basic safety needs and physical care needs are anticipated within inherent dignity. Please clarify whether these needs are based on a specific theoretical framework such as Maslow or a nursing theory. If not, consider adding a brief reference.

Two, you stated that personal factors such as functional status, organizational factors such as ethical care environment, and sociocultural factors such as collectivism or ageism may influence dignity. This is good but please provide at least one reference for each category of factors to strengthen your argument.

Three, you compared your expected findings to previous reviews and cited references 37, 38, and 39. However you did not specify what these previous reviews found and how your review will differ. Please add one or two sentences explaining the specific gaps in those previous reviews that your review will address.

Four, you mentioned challenges in synthesizing themes that accurately represent original findings. This is a valid concern. However you did not mention how you will handle conflicting findings across studies. Please add a brief statement about how disagreements in synthesized themes will be resolved.

Five, you stated that future research should incorporate multiple demographic perspectives. Please specify which demographics you mean, for example age subgroups, gender, or cultural backgrounds.

Limitations

This section is honest and well written. However I have several comments.

One, you acknowledged language bias and the use of DeepL translation. This is good. However you did not mention the possibility that some non-English and non-Chinese studies might be missed entirely if they are not indexed in your selected databases. Please add this.

Two, you acknowledged exclusion of grey literature and the risk of publication bias. This is good. However you did not mention the possibility that excluding theses and dissertations may miss emerging research from early career researchers. Please consider adding this point.

Three, you stated that findings may not be fully transferable to other cultural contexts given Chinese cultural values. This is a valid limitation. However you did not mention that findings may also not be transferable to other Asian cultures such as Japanese or Korean which have different aging and long-term care systems. Please add this.

Four, you did not mention the risk of reviewer bias in coding and theme development even though two reviewers will extract data independently. Please add this as a limitation because no matter how independent the reviewers are, their interpretations may still be influenced by their own cultural and professional backgrounds.

Five, you did not mention that the search will be conducted at a single point in time and any studies published after August 2025 will not be included. This is a standard limitation for any systematic review. Please add it.

Conclusions

This section is well written. However I have several comments.

One, you stated that this review aims to synthesize qualitative evidence including Chinese literature. This is already stated in your aim. Consider making your conclusion more impactful by stating the expected clinical or policy implications explicitly rather than repeating the aim.

Two, you recommended future research to explore dignity from perspectives of nursing staff, family members, administrators, and policymakers. This is good. However you did not mention the perspective of the older adults themselves which you already included. Please rephrase to avoid confusion.

Reference

One, reference number 1 is from 2026. You are writing this systematic review protocol in 2024 or 2025. A 2026 publication cannot exist at the time you are writing your protocol. Please check the year. If this is a future publication, you cannot cite it. If the year is a typo, please correct it to the actual publication year.

Two, reference number 25 is DeepL Translator 2026. Same issue. The year 2026 has not arrived yet. Please correct to the actual version year or the year you accessed the tool.

Three, reference number 3 is WHO 2015. This is a valid reference. However the WHO has more recent reports on ageing and health such as the Decade of Healthy Ageing 2021-2030 reports. Consider adding more recent WHO sources.

Four, reference number 16 is the Universal Declaration of Human Rights 1948. This is correct. However you are citing this as a source for inherent dignity. Consider also citing more recent commentaries on human dignity and ageing.

Five, reference number 20 is PRISMA-P 2015. This is correct for your protocol. However ensure you also cite PRISMA 2020 for the review itself. You mentioned PRISMA 2020 in your methods but I do not see it cited separately. Please add reference 21 as PRISMA 2020 statement. You have it but check the citation format.

Six, reference number 31 is Hannes et al 2010. This is a comparative analysis of appraisal tools. However this study is 14 years old. Consider adding more recent evidence comparing JBI with other tools.

Seven, references 32 and 33 are about AMSTAR. You are using JBI, not AMSTAR. Why are you citing AMSTAR validation studies? These references seem irrelevant to your JBI-based quality appraisal. Please either justify why these are included or remove them.

Eight, reference number 37 is Wachholz and Giacomin 2022. This is a commentary piece from F1000Research. Please verify that this is a peer-reviewed source appropriate for your discussion.

Nine, reference number 38 is Šaňáková and Čáp 2019. This is a qualitative literature review. It is appropriate. No issue.

Ten, reference number 39 is Hall et al 2014. This is ten years old at the time of your review. Consider adding more recent studies on dignity from multiple perspectives.

[Note: HTML markup is below. Please do not edit.]

To ensure your figures meet our technical requirements, please review our figure guidelines: https://journals.plos.org/plosone/s/figures

You may also use PLOS’s free figure tool, NAAS, to help you prepare publication quality figures: https://journals.plos.org/plosone/s/figures#loc-tools-for-figure-preparation.

NAAS will assess whether your figures meet our technical requirements by comparing each figure against our figure specifications.

# Author comment: Author response to Decision Letter 2

**DOI:** 10.1371/journal.pone.0351774.r004

10 May 2026

Reviewer comments Response Location in MS Type(Major or Minor)

1 If you would like to make changes to your financial disclosure, please include your updated statement in your cover letter. Guidelines for resubmitting your figure files are available below the reviewer comments at the end of this letter. (1)No need to revise the financial disclosure.

(2)Last version we have revised the figures use free NAAS figure tool of PLOS.

2 If applicable, we recommend that you deposit your laboratory protocols in protocols.io to enhance the reproducibility of your results. Protocols.io assigns your protocol its own identifier (DOI) so that it can be cited independently in the future. For instructions see: https://journals.plos.org/plosone/s/submission-guidelines#loc-laboratory-protocols. Additionally, PLOS ONE offers an option for publishing peer-reviewed Lab Protocol articles, which describe protocols hosted on protocols.io. Read more information on sharing protocols at https://plos.org/protocols?utm_medium=editorial-email&utm_source=authorletters&utm_campaign=protocols.

Our manuscript employs a qualitative synthesis methodology and does not involve laboratory protocols. Therefore, depositing on protocols.io is not applicable to our study.

3 If the reviewer comments include a recommendation to cite specific previously published works, please review and evaluate these publications to determine whether they are relevant and should be cited. There is no requirement to cite these works unless the editor has indicated otherwise. We don’t have cited the previously published works.

4 Please review your reference list to ensure that it is complete and correct. If you have cited papers that have been retracted, please include the rationale for doing so in the manuscript text, or remove these references and replace them with relevant current references. Any changes to the reference list should be mentioned in the rebuttal letter that accompanies your revised manuscript. If you need to cite a retracted article, indicate the article’s retracted status in the References list and also include a citation and full reference for the retraction notice. We have carefully reviewed our reference list for completeness and accuracy. No retracted articles were identified among our citations, and no changes to the reference list were made.

Additional Editor Comments

Abstract: Your abstract is well written but three essential elements are missing. Please add the following to your abstract before resubmission.

One. Please specify the search timeframe. You need to state the period of the search, for example from inception to 2024 or from 2000 to 2024. Add the timeframe from inception to 31th August 2025. Line 18-19 Minor

Two. Please specify your inclusion and exclusion criteria. You need to state the language criteria such as English and Chinese only. You also need to state whether grey literature such as conference proceedings and dissertations will be included or excluded. We have added the inclusion and exclusion criteria to the abstract as requested:

Language criteria: No language restrictions will be applied (we include all languages, not only English and Chinese).

Grey literature: conference proceedings, dissertations, theses, reports will be excluded. Line 19-25 Major

Three. Please add transparency and reproducibility elements. We have added transparency and reproducibility elements to the abstract as requested. This system review will follow the PRISMA 2020 guidelines and will be registered with PROSPERO.

Three reviewers will independently screen, two professional reviewers extract data, and assess study quality.

Disagreements will be resolved by a third expert. JBI critical appraisal tools will help to assess the quality of each article.

Full search strategies will be provided as supplementary materials. Line26-33 Major

Introduction: Your introduction is well written and has several strengths. The problem is clearly stated, the aim is specific, the justification for the review is clear, the theoretical framework of Jacobson and Høy is properly introduced, the Chinese cultural context is explicitly addressed, and the gap in literature is well identified.

One.

Please check your in-text citations. Some numbers appear without corresponding references. Ensure every citation number has a full reference in your reference list. Thank you for your comment on the in text citations.

Throughout the manuscript, when multiple consecutive references are cited, they are presented as a range (e.g., [3-6] instead of [3,4,5,6]).

These ranges include all numbers in between. Every number appearing in any such range has a full and matching reference in the reference list.

I have double checked the entire document, and no citation number is missing its corresponding reference. Minor

Two. Your first paragraph is very long. Consider breaking it into two shorter paragraphs for better readability. We break the first paragraph into two shorter paragraphs: First shorter paragraph introduces the phenomenon of aging in China and nursing home is a necessary choice for older adults. Second paragraph introduces the existing caring problems in nursing home. Line48-64 Minor

Three. You wrote "hybrid analytical approach" in the last paragraph but you did not explain what this means. Please clarify this term or remove it if not necessary. Remove the hybrid analytical approach. Line89. Minor

Four. You stated that this review will use meta-aggregation and then examine findings through Jacobson's framework. Please clarify whether the framework will guide the synthesis or will be applied after the synthesis. needs to be explicit. The framework just used for the categorizing the themes after the synthesis and help to identify the domains. Line 89-98 Major

Method: Methods section is strong and well developed. The ten issues identified need to be corrected.

One. Date inconsistency. You wrote "31st September 2025" as your search completion date. September has 30 days, not 31. Please correct to 30th September 2025. The search covered the period from 1966 to 31 August 2025.And the search completed data is 30th September 2025, so I revise the data to keep consistency. Line178 Minor

Two. Year inconsistency in timeline. In your timeline you wrote October 1-October 31, 2025 and November 1-November 30, 2025 but your search completion date is August 31, 2025. These dates do not match. Please revise your timeline to be consistent with your search completion date. We would like to respectfully clarify that August 31, 2025 refers to the search end date(the date limit applied to the database search), while September 30, 2025 is the search completion date (the date by which the search will be fully executed and recorded), as noted in your previous comment. Therefore, the October and November phases are fully consistent with the search completion date of September 30, 2025. To avoid any further confusion, we have added explicit labels in the timeline to clearly distinguish the search end date from the search completion date. Line 346-356 Major

Three. Missing justification for excluding grey literature. You excluded theses, dissertations, and non-peer-reviewed materials. This is a major limitation that may introduce publication bias. Please provide a clear rationale for why grey literature is excluded. We describe the reason for excluding the grey literature. Line 157-161 Minor

Four Missing explanation for excluding older adults with Alzheimer's disease. You excluded older adults with severe Alzheimer's or psychological disease. Please explain why this population is excluded as their dignity is also an important concern. In the current study, we excluded this population primarily for methodological and ethical reasons related to informed consent and response capability. Line 136-138

Minor

Five. Translation method issue. You stated you will use DeepL for non-English and non-Chinese literature. Machine translation may miss nuanced meanings of dignity. Please add that you will have a bilingual expert verify all translated content. We would like to clarify that this step has already been included in our manuscript. As stated in the [Methods/Translation section], all translated content from non-English and non-Chinese literature will be initially processed by DeepL and then independently verified by a bilingual expert to ensure the accurate interpretation of nuanced concepts such as "dignity." We have highlighted this in the revised manuscript for clarity. Line 231-232 Minor

Six. PRISMA version inconsistency. You cited PRISMA 2015 for the protocol and PRISMA 2020 for the review. Please use PRISMA 2020 for both or clarify why different versions are used. Thank you for your valuable comment. We would like to clarify that different reporting guidelines are applied at different stages of a systematic review.

This study is a systematic review protocol,so its report is in accordance with the PRISMA-P 2015 statement, which is specifically developed for systematic review protocols and has not yet been updated to a 2020 version.

For the completed systematic review in the future, we will follow the PRISMA 2020 guidelines, which represent the most recent standard for reporting systematic reviews.

Therefore, the use of PRISMA-P 2015 for the protocol and PRISMA 2020 for the final review is appropriate and consistent with current methodological recommendations. Line119-121 Minor

Seven. Missing sample of search strategy. You provided Table 2 but did not show the actual search string with Boolean operators. Please provide an example of the full search strategy for one database such as PubMed. We have now added a full example of the search strategy for PubMed in the Methods section (Search strategy subsection). The example includes all Boolean operators (AND, OR), Mesh terms(aged), truncation (digni*), and the date restriction (from 1966 01 January to 31 August 2025). Line179-188 Minor

Eight. Author roles are unclear. You mentioned a researcher, an assistant graduate student, and a non-nursing assistant. Their qualifications are not described. Please clarify who these individuals are and their level of training. We have described the roles of all the team members and described their level of training. Line189-204 Major

Nine. JBI cutoff scores need justification. You set 80% for high quality and 50% for low quality. Please provide a reference or justification for these specific cutoff scores. We cited the reference and described the justification for the cutoff score. Line259-267 Minor

Ten. Low quality studies decision is vague. You stated low quality studies will be "reviewed and discussed" with a decision to retain or exclude. This is subjective. Please specify clear criteria for when a low quality study will be retained versus excluded. According to the JBI manual of systematic review,we added an exclusion criteria. Line267-278 Major

Discussion: This section is well written. However I have several comments.

One. You stated that themes related to basic safety needs and physical care needs are anticipated within inherent dignity. Please clarify whether these needs are based on a specific theoretical framework such as Maslow or a nursing theory. If not, consider adding a brief reference. We agree with your comments and have added Maslow’s Hierarchy of Needs Theory to underpin the themes related to basic safety and physical care needs within inherent dignity. Line363-366 Minor

Two. You stated that personal factors such as functional status, organizational factors such as ethical care environment, and sociocultural factors such as collectivism or ageism may influence dignity. This is good but please provide at least one reference for each category of factors to strengthen your argument.

Follow your comments we have added the reference for each factor to enhance the credibility. Line367-373 Minor

Three. You compared your expected findings to previous reviews and cited references 37, 38, and 39. However you did not specify what these previous reviews found and how your review will differ. Please add one or two sentences explaining the specific gaps in those previous reviews that your review will address. Thank you for this constructive suggestion. We agree that the original text lacked specificity regarding the limitations of the cited reviews. We have revised the manuscript accordingly by adding two sentences to clarify the gaps in the previous reviews and how our review will address them. Line 382-386 Minor

Four. You mentioned challenges in synthesizing themes that accurately represent original findings. This is a valid concern. However you did not mention how you will handle conflicting findings across studies. Please add a brief statement about how disagreements in synthesized themes will be resolved. We agree that addressing how conflicting findings across studies will be handled is essential for methodological transparency. In response, we have added a sentence to the clarify that conflicting findings. Line397-402 Minor

Five. You stated that future research should incorporate multiple demographic perspectives. Please specify which demographics you mean, for example age subgroups, gender, or cultural backgrounds. Follow your advice, we add the specific information in the demographics. Line 403-412 Major

Limitations: This section is honest and well written. However I have several comments.

One. You acknowledged language bias and the use of DeepL translation. This is good. However you did not mention the possibility that some non-English and non-Chinese studies might be missed entirely if they are not indexed in your selected databases. Please add this. Follow your comments, we added this item in our limitation. Line 418-419 Minor

Two You acknowledged exclusion of grey literature and the risk of publication bias. This is good. However you did not mention the possibility that excluding theses and dissertations may miss emerging research from early career researchers. Please consider adding this point. We agree that it is an important limitation that warrants explicit acknowledgement. We have revised the sentence accordingly to include this point. Line423-425 Minor

Three You stated that findings may not be fully transferable to other cultural contexts given Chinese cultural values. This is a valid limitation. However you did not mention that findings may also not be transferable to other Asian cultures such as Japanese or Korean which have different aging and long-term care systems. Please add this. We agree that specifying the limitation of transferability even within Asian cultural contexts strengthens the rigor of our discussion. We have added a sentence acknowledging that the findings may not be directly applicable to other Asian contexts. Line 429-432 Minor

Four. You did not mention the risk of reviewer bias in coding and theme development even though two reviewers will extract data independently. Please add this as a limitation because no matter how independent the reviewers are, their interpretations may still be influenced by their own cultural and professional backgrounds. We agree that reviewer bias and we added this point in the limitation section. Line 432-439 Minor

Five. You did not mention that the search will be conducted at a single point in time and any studies published after August 2025 will not be included. This is a standard limitation for any systematic review. Please add it. This is an important point in the systematic review. Follow this comment, we add this standard limitation in the limitation section. Line 439-441 Minor

Conclusions: This section is well written. However I have several comments.

One. You stated that this review aims to synthesize qualitative evidence including Chinese literature. This is already stated in your aim. Consider making your conclusion more impactful by stating the expected clinical or policy implications explicitly rather than repeating the aim.

In the revised manuscript, we have removed the restatement of the aim and replaced it with explicit clinical and policy implications drawn from our findings. Line448-451

Line460-467 Major

Two. You recommended future research to explore dignity from perspectives of nursing staff, family members, administrators

Attachment Submitted filename: Response_to_Reviewers_auresp_2.docx

# Editor report: Decision Letter 2

**DOI:** 10.1371/journal.pone.0351774.r005

**Authors:** Samar Sobhi

15 May 2026

PONE-D-25-46837R2

The perception and experience of dignity in the care of older adults in nursing homes: A Meta-aggregation protocol

PLOS One

Dear Dr. Li,

Thank you for submitting your manuscript to PLOS ONE. After careful consideration, we feel that it has merit but does not fully meet PLOS ONE’s publication criteria as it currently stands. Therefore, we invite you to submit a revised version of the manuscript that addresses the points raised during the review process.

Please submit your revised manuscript by Jun 29 2026 11:59PM. If you will need more time than this to complete your revisions, please reply to this message or contact the journal office at plosone@plos.org. When you're ready to submit your revision, log on to https://www.editorialmanager.com/pone/ and select the 'Submissions Needing Revision' folder to locate your manuscript file.

Please include the following items when submitting your revised manuscript:

- A letter that responds to each point raised by the academic editor and reviewer(s). You should upload this letter as a separate file labeled 'Response to Reviewers'.
- A marked-up copy of your manuscript that highlights changes made to the original version. You should upload this as a separate file labeled 'Revised Manuscript with Track Changes'.
- An unmarked version of your revised paper without tracked changes. You should upload this as a separate file labeled 'Manuscript'.

If you would like to make changes to your financial disclosure, please include your updated statement in your cover letter. Guidelines for resubmitting your figure files are available below the reviewer comments at the end of this letter.

If applicable, we recommend that you deposit your laboratory protocols in protocols.io to enhance the reproducibility of your results. Protocols.io assigns your protocol its own identifier (DOI) so that it can be cited independently in the future. For instructions see: https://journals.plos.org/plosone/s/submission-guidelines#loc-laboratory-protocols. Additionally, PLOS ONE offers an option for publishing peer-reviewed Lab Protocol articles, which describe protocols hosted on protocols.io. Read more information on sharing protocols at https://plos.org/protocols?utm_medium=editorial-email&utm_source=authorletters&utm_campaign=protocols.

As the corresponding author, your ORCID iD is verified in the submission system and will appear in the published article. PLOS supports the use of ORCID, and we encourage all coauthors to register for an ORCID iD and use it as well. Please encourage your coauthors to verify their ORCID iD within the submission system before final acceptance, as unverified ORCID iDs will not appear in the published article. *Only*  the individual author can complete the verification step; PLOS staff *cannot*  verify ORCID iDs on behalf of authors.

We look forward to receiving your revised manuscript.

Kind regards,

Samar Sobhi

Guest Editor

PLOS One

Journal Requirements:

1. If the reviewer comments include a recommendation to cite specific previously published works, please review and evaluate these publications to determine whether they are relevant and should be cited. There is no requirement to cite these works unless the editor has indicated otherwise.

2. Please review your reference list to ensure that it is complete and correct. If you have cited papers that have been retracted, please include the rationale for doing so in the manuscript text, or remove these references and replace them with relevant current references. Any changes to the reference list should be mentioned in the rebuttal letter that accompanies your revised manuscript. If you need to cite a retracted article, indicate the article’s retracted status in the References list and also include a citation and full reference for the retraction notice.

Additional Editor Comments:

Abstract:

· Shorten the aim. Example: "This systematic review aims to conceptualize the characteristics of dignity in nursing home care for older adults, with attention to Chinese cultural perspectives."

· Reword reviewer roles for clarity: "Three reviewers will screen titles and abstracts. Two reviewers will then independently select studies and extract data using predesigned forms. Any discrepancies will be documented and resolved by a third reviewer until consensus is reached."

· Remove redundant "assistance... assist" – use "a third reviewer will resolve discrepancies".

· Change "will be clearly claimed" to "will be documented"

The introduction is well‑written and covers the necessary background. No revisions needed.

Methods Section:

The overall structure is clear and follows standard systematic review protocol guidelines. However, a few minor revisions are needed:

· Correct typo: change "system review" to "systematic review".

· Minor language fix: change "older adults live in nursing homes" to "older adults living in nursing homes".

· Grey literature justification is good but could be shortened (optional).

1. Typo in Table title – "Seach Strategy" should be "Search Strategy" (Table 2).

2. Redundant sentence – In the first paragraph, you have: "The search covered the period from 1966 to 31 August 2025" – this is already stated twice (once with database inception, once at the end). Remove the second mention.

3. Reviewer roles – Clarify who does what. You describe 3 reviewers with different backgrounds, but then state that study selection and critical appraisal are performed by two reviewers (primary researcher + nursing graduate student). The non‑nursing assistant does initial screening only. This is fine, but ensure consistency in the description.

4. Typo – "assitantant" should be "assistant" (in reviewer team description).

5. Minor language – "will be jointly responsible" → "will be jointly responsible" (remove extra "s" in "jointly"? already correct). "cannot be available" → "are not available".

6. Low‑quality study exclusion – Your plan is good (retain by default, exclude only if critical flaw). However, the sentence is long. Consider shortening for clarity.

7. Missing PROSPERO registration number – As noted earlier, recommended for transparency.

8. Timeline typo – "Januray" should be "January" , "Februray" should be "February" .

The discussion section is well‑written, comprehensive, and appropriately linked to the theoretical framework. No major revisions are needed.

These final sections are well‑written and require only a few minor correction:

Change "the study will exclude" to "this review will exclude" .

· Simplify "there lies a potential interpretive bias" to "interpretive bias is a potential limitation" .

List of Abbreviations

· Correct "Phenomenonon of interest" to "Phenomenon of interest" .

Support Information

· Correct "S2 Table. Table 2 Search Strategy" to "S2 Table. Search Strategy" (remove duplicate "Table 2")

The reference list is comprehensive. A few minor corrections are needed:

· Reference 15: Incomplete DOI. Please correct or remove it.

· Reference 29: Empty DOI placeholder (https://doi.org/https://doi.org/). Please remove or provide the correct DOI

[Note: HTML markup is below. Please do not edit.]

[NOTE: If reviewer comments were submitted as an attachment file, they will be attached to this email and accessible via the submission site. Please log into your account, locate the manuscript record, and check for the action link "View Attachments". If this link does not appear, there are no attachment files.]

To ensure your figures meet our technical requirements, please review our figure guidelines: https://journals.plos.org/plosone/s/figures

You may also use PLOS’s free figure tool, NAAS, to help you prepare publication quality figures: https://journals.plos.org/plosone/s/figures#loc-tools-for-figure-preparation.

NAAS will assess whether your figures meet our technical requirements by comparing each figure against our figure specifications.

# Author comment: Author response to Decision Letter 3

**DOI:** 10.1371/journal.pone.0351774.r006

24 May 2026

No

Attachment Submitted filename: Response_to_Reviewers_auresp_3.docx

# Editor report: Decision Letter 3

**DOI:** 10.1371/journal.pone.0351774.r007

**Authors:** Samar Sobhi

2 Jun 2026

The perception and experience of dignity in the care of older adults in nursing homes: A Meta-aggregation protocol

PONE-D-25-46837R3

Dear Dr. Li,

We’re pleased to inform you that your manuscript has been judged scientifically suitable for publication and will be formally accepted for publication once it meets all outstanding technical requirements.

Within one week, you’ll receive an e-mail detailing the required amendments. When these have been addressed, you’ll receive a formal acceptance letter and your manuscript will be scheduled for publication.

An invoice will be generated when your article is formally accepted. Please note, if your institution has a publishing partnership with PLOS and your article meets the relevant criteria, all or part of your publication costs will be covered. Please make sure your user information is up-to-date by logging into Editorial Manager at Editorial Manager® and clicking the ‘Update My Information' link at the top of the page. For questions related to billing, please contact billing support.

If your institution or institutions have a press office, please notify them about your upcoming paper to help maximize its impact. If they’ll be preparing press materials, please inform our press team as soon as possible -- no later than 48 hours after receiving the formal acceptance. Your manuscript will remain under strict press embargo until 2 pm Eastern Time on the date of publication. For more information, please contact onepress@plos.org.

Kind regards,

Samar Sobhi

Guest Editor

PLOS One

Additional Editor Comments (optional):

The manuscript is methodologically sound and addresses an important topic. However, minor editorial revisions are needed before acceptance. Please correct the following: remove redundant phrases, shorten overly long sentences, fix citation style consistency (use numbers only), and break long paragraphs into shorter ones for better readability. No changes to the scientific content or methodology are required.

Reviewers' comments:

# Editor report: Acceptance letter

**DOI:** 10.1371/journal.pone.0351774.r008

**Authors:** Samar Sobhi

PONE-D-25-46837R3

PLOS One

Dear Dr. Li,

I'm pleased to inform you that your manuscript has been deemed suitable for publication in PLOS One. Congratulations! Your manuscript is now being handed over to our production team.

At this stage, our production department will prepare your paper for publication. This includes ensuring the following:

* All references, tables, and figures are properly cited

* All relevant supporting information is included in the manuscript submission,

* There are no issues that prevent the paper from being properly typeset

You will receive further instructions from the production team, including instructions on how to review your proof when it is ready. Please keep in mind that we are working through a large volume of accepted articles, so please give us a few days to review your paper and let you know the next and final steps.

Lastly, if your institution or institutions have a press office, please let them know about your upcoming paper now to help maximize its impact. If they'll be preparing press materials, please inform our press team within the next 48 hours. Your manuscript will remain under strict press embargo until 2 pm Eastern Time on the date of publication. For more information, please contact onepress@plos.org.

You will receive an invoice from PLOS for your publication fee after your manuscript has reached the completed accept phase. If you receive an email requesting payment before acceptance or for any other service, this may be a phishing scheme. Learn how to identify phishing emails and protect your accounts at https://explore.plos.org/phishing.

If we can help with anything else, please email us at customercare@plos.org.

Thank you for submitting your work to PLOS ONE and supporting open access.

Kind regards,

PLOS ONE Editorial Office Staff

on behalf of

Dr. Samar Sobhi

Guest Editor

PLOS One
